# A modeling framework for detecting and leveraging node-level information in Bayesian network inference

**DOI:** 10.1093/biostatistics/kxae021

**Published:** 2024-06-25

**Authors:** Xiaoyue Xi, Hélène Ruffieux

**Affiliations:** MRC Biostatistics Unit, University of Cambridge, East Forvie Building, Forvie Site, Robinson Way, Cambridge CB2 0SR, United Kingdom; MRC Biostatistics Unit, University of Cambridge, East Forvie Building, Forvie Site, Robinson Way, Cambridge CB2 0SR, United Kingdom

**Keywords:** Bayesian hierarchical model, Gaussian graphical model, gene expression network, node-level auxiliary variables, sparse precision matrices, spike-and-slab prior, variable selection, variational inference

## Abstract

Bayesian graphical models are powerful tools to infer complex relationships in high dimension, yet are often fraught with computational and statistical challenges. If exploited in a principled way, the increasing information collected alongside the data of primary interest constitutes an opportunity to mitigate these difficulties by guiding the detection of dependence structures. For instance, gene network inference may be informed by the use of publicly available summary statistics on the regulation of genes by genetic variants. Here we present a novel Gaussian graphical modeling framework to identify and leverage information on the centrality of nodes in conditional independence graphs. Specifically, we consider a fully joint hierarchical model to simultaneously infer (i) sparse precision matrices and (ii) the relevance of node-level information for uncovering the sought-after network structure. We encode such information as candidate auxiliary variables using a spike-and-slab submodel on the propensity of nodes to be hubs, which allows hypothesis-free selection and interpretation of a sparse subset of relevant variables. As efficient exploration of large posterior spaces is needed for real-world applications, we develop a variational expectation conditional maximization algorithm that scales inference to hundreds of samples, nodes and auxiliary variables. We illustrate and exploit the advantages of our approach in simulations and in a gene network study which identifies hub genes involved in biological pathways relevant to immune-mediated diseases.

## 1 Introduction

Undirected graphs are useful tools for expressing relationships between random variables. They are depicted as undirected diagrams where nodes represent variables, and edges represent conditional dependence between nodes, given the remaining nodes in the graph (*partial correlation*). The presence of an edge between two nodes therefore indicates that the corresponding variables are *directly* associated, which provides natural understanding of relationships between variables in many practical applications. Gaussian graphical models provide a framework for estimating such relationships by modeling the variables using a multivariate Gaussian distribution with sparse precision matrix. In this setting, a zero entry in the precision matrix is equivalent to a zero partial correlation between the corresponding two Gaussian random variables, that is, the absence of an edge between the nodes. This effectively reduces graph estimation to recovering the support of the precision matrix.

Most existing Gaussian graphical models estimate precision matrices from independent and identically distributed samples of a random vector $ (Y_{1},\ldots,Y_{P})$, by treating the nodes *Y_j_*, $ j=1,\ldots,P$, as a priori exchangeable (Yuan and Lin 2007; Friedman et al. 2008; Wang 2012; Li and McCormick 2019). However, this assumption is often unrealistic, especially when exogenous factors are thought to influence (some of) the nodes and, therefore, the network dependence structure as a whole. In such cases, node-level auxiliary variables may provide additional information on the importance (or “centrality” or propensity to have high degree) of each node in the graph structure.

This idea has been used in the context of regression models to improve variable selection, estimation of regression effects and prediction by encoding predictor-level auxiliary variables. Notably, van de Wiel et al. (2016) introduced the concept of “co-data” to formalize the task of exploiting external information on predictors, and Novianti et al. (2017) and Ruffieux et al. (2021) proposed further development for high-dimensional regression models, with applications in genomic studies with auxiliary annotation variables (epigenetic marks, probe grouping, conservation status of microRNAs) obtained independently of the outcome. More recently, Li and McCormick (2019) and Bu and Lederer (2021) leveraged edge-wise knowledge, such as the similarity between node attributes, to facilitate edge identification in network estimation. Jewson et al. (2022) proposed a graphical lasso framework that exploits auxiliary networks, obtained from external information, to guide inference about the network of primary interest. However, to the best of our knowledge, no graphical modeling approach permits directly accommodating node-level auxiliary variables to aid the detection and interpretation of partial dependence structures. Importantly, such an endeavor differs from directly modifying the prior probabilities of nodes to have high (or low) degree based on some pre-established expert knowledge. Indeed, by *estimating* the effects of candidate node-level variables on the graph structure, their relevance and influence on the nodes are *inferred* from the data in an agnostic fashion. Such a framework may thus improve the estimation of graph structures while enabling the discovery of mechanisms driving these structures. This is particularly relevant for biological networks, which are expected to be *scale-free* (Khanin and Wit 2006)—ie most nodes have a relatively low degree, while a few nodes, known as “hubs”, have a high degree—so that node attributes might be leveraged to inform the propensity of nodes to be hubs.

The high dimensionality of graphs, inherent to the quadratic relationship between the number of nodes and the number of parameters, requires enforcing sparsity on the off-diagonal entries of the precision matrix. To achieve this, frequentist methods use regularization techniques based, e.g. on the lasso penalty (Yuan and Lin 2007; Friedman et al. 2008) or the ridge penalty (Krämer et al. 2009). Bayesian methods achieve sparsity using shrinkage priors, typically relying on Markov chain Monte Carlo (MCMC) inference. In particular, Gibbs samplers have been developed for graphical models under double exponential (Wang 2012), spike-and-slab (Wang 2015) and horseshoe (Li et al. 2019a) priors. However, stochastic search algorithms become computationally inefficient in most real-world problems where the number of nodes exceeds a few tens. To address this, Li and McCormick (2019) subsequently proposed an expectation conditional maximization (ECM) algorithm as a faster, deterministic alternative to sampling-based inference for a spike-and-slab graphical model. Yet, ensuring scalable inference while allowing for uncertainty quantification for parameters of interest remains difficult in graphical settings.

We tackle the aforementioned challenges by introducing a Bayesian hierarchical framework with contributions at the modeling and inference stages. First, we propose a fully joint two-level spike-and-slab model that exploits a (possibly) large set of node-level candidate auxiliary variables to estimate the graph structure, while inferring probabilities of informativeness, for each variable, about this structure. Such a formulation is beneficial from both accuracy and interpretability standpoints. Indeed, *inferring* the subset of auxiliary variables associated with the graph structure should improve the estimation of the adjacency matrix and, in turn, that of the precision matrix, by ensuring that only the variables relevant to this estimation are leveraged, with no *ad-hoc* preselection. Moreover, inspecting the global influence of the retained variables on the centrality of nodes may also offer practitioners valuable insights into the exogenous factors in play and their possible role in shaping the network structure. Second, we develop a novel variational Bayes expectation conditional maximization (VBECM) algorithm that scales comparably to pure ECM algorithms but approximates full posterior distributions rather than point estimates.

Importantly, our work is concerned with accounting for node-level information, rather than sample-level information to enhance estimation of graphs. While the latter goal has recently been studied through the formulation of covariate-dependent Gaussian graphical models (Ni et al. 2022; Zhang and Li 2022), developing efficient approaches—both statistically and computationally—to tackle the former goal is at least as important, given the wealth of external annotations that are now collected alongside datasets of primary interest. As hinted above, this is for instance the case of molecular datasets, for which complementary databases are growing in size and diversity (e.g. about epigenetic mechanisms, gene function and regulation). We will illustrate our framework in a monocyte gene network problem, exploiting summary statistics about the control of genes by genetic variants. Of course, the applicability of our framework extends beyond the field of molecular biology, as the model is generic and free from any domain-specific assumptions.

This article is organized as follows. Section 2 introduces the monocyte gene expression problem and discusses the potential benefits of encoding gene-level auxiliary information about the genetic control of genes in the network. Section 3 recalls the classical Gaussian graphical model with spike-and-slab prior upon which our approach is based, and details our hierarchical model to select and leverage node-level auxiliary variables. Section 4 describes our VBECM inference algorithm. Section 5 evaluates the statistical and computational performance of our approach in a series of simulations designed to emulate real data settings. Section 6 applies and exploits our framework on the monocyte data, compares it with a classical Gaussian graphical modeling approach (ie with no use of auxiliary information), and discusses potential biological implications of our findings in the context of immune-mediated diseases. Section 7 summarizes our work, highlights further application domains and suggests future methodological developments.

## 2 Data and motivating example

We introduce a gene expression dataset to discuss the benefits of using relevant annotations for guiding gene network inference and motivate the development of our framework to do so. The data consist of gene expression from 432 European individuals, quantified from CD14$^{+}$ monocytes using Illumina HumanHT-12 v4 BeadChip arrays (Fairfax et al. 2012, 2014). Monocytes are myeloid innate immune cells, which play a crucial role in host defence and inflammation by initiating cytokine-mediated response upon microorganism invasion. Therefore, monocytes provide useful clues to investigate disease processes, evaluate potential therapeutic targets and, ultimately, develop novel treatment strategies (Ma et al. 2019).

Verdugo et al. (2013) analyzed monocyte gene levels using co-expression networks and uncovered molecular mechanisms likely underlying atherosclerosis in smokers. The authors estimated indirect relationships (marginal correlation), whereas our focus is on estimating direct relationships (partial correlation). Conditional independence networks allow pinpointing direct dependencies between genes, and are thus well suited to highlight groups of genes forming signaling pathways, or “regulatory programs”, that are activated in disease conditions (Schäfer and Strimmer 2005). For instance, Bayesian networks have been used to examine the disruption and conservation of gene pathways during chronic obstructive pulmonary disease (Shaddox et al. 2018) and multiple myeloma (a late-stage bone marrow malignancy; Ni et al. 2022).

None of the above studies used auxiliary annotations and yet, when adequately factored in, the growing diversity of annotation sources on gene and protein levels offers numerous possibilities for refining the detection of dependence structures. Such annotations include summary statistics on the regulation of genes by genetic variants (Ruffieux et al. 2020; Kerimov et al. 2021), information on biological pathway membership (Kanehisa and Goto 2000; Ashburner et al. 2000) or scores on gene-level epigenetic activity (Budden and Crampin 2016), to list a few.

Here we propose to capitalize on information about genetic regulation in monocytes to guide the estimation of network dependence structures. Specifically, major *hotspots* (ie genetic variants regulating a large number of nearby and remote genes) have been observed on chromosome 12 in previous monocyte studies (Fairfax et al. 2014; Momozawa et al. 2018), and there is evidence that genes regulated by a same hotspot are more likely to be functionally related and thus to share edges in the network (Van Dyke et al. 2021). Those in the vicinity of the hotspot also tend to be more tightly controlled by it, and may mediate its effect on other remote genes. In the network, such mediating genes are typically more connected to other genes and form hubs (“central” nodes, with high degrees). Thus, accommodating summary statistics on the control of genes by hotspots may aid in uncovering gene dependence patterns including gene hubs. In addition, immune stimulation of CD14$^{+}$ monocytes, such as through exposure to inflammatory proxies interferon-*γ* (IFN-*γ*), tends to trigger enhanced regulatory activity, potentially leading to the formation of additional genetic hotspots (Kim et al. 2014; Fairfax et al. 2014) and, as a result, stronger hub patterns in the network.

In this paper, we will construct gene-level auxiliary variables from posterior probabilities of association between genetic variants and genes (our summary statistics) obtained from a genome-wide association study performed with the joint mapping approach *atlasqtl* (Ruffieux et al. 2020) using independent genetic and CD14$^{+}$ monocyte gene expression data (Momozawa et al. 2018). Most of these probabilities are close to zero, reflecting the fact that genetic variants typically control only a few genes, if any. We will use these summary statistics, in the context of two network estimation problems, namely for genes quantified from resting monocytes as well as IFN-*γ*-stimulated monocytes. By encoding information about the genetic regulation of those genes, we hope to not only to improve network inference, but also to suggest genetic variants possibly triggering gene dependence structures, thanks to the hypothesis-free selection of auxiliary variables that is built into our hierarchical spike-and-slab framework. Efficient inference is a prerequisite to applying our approach to the hundreds of genes, samples and tens of candidate auxiliary variables represented in our data. This corresponds to dimensions encountered in many other real-world scenarios, whereby sampling methods, based on MCMC inference, would be computationally prohibitive. The VBECM procedure we will develop is aimed at producing scalable yet accurate inference, with approximations of full posterior distributions. We will return to the monocyte problem in Section 6.

## 3 Methodology

### 3.1 The spike-and-slab graphical model

We consider a Gaussian graphical model with *N* observations on *P* nodes. The data are represented by a matrix $ \boldsymbol{Y}\in \mathbb{R}^{N\times P}$ whose rows $ \boldsymbol{y}_{\boldsymbol{n}},\,n=1,\ldots,N$, are independent and identically distributed samples from a multivariate Gaussian distribution, ie

(1) $$\displaystyle \boldsymbol{y}_{\boldsymbol{1}},\ldots,\boldsymbol{y}_{\boldsymbol{N}}\overset{\mathrm{iid}}{\sim}\mathcal{N}_{P}\left(\boldsymbol{\mu},\mathbf{\Omega}^{-1}\right),\quad \mathbf{\Omega}\in \mathcal{M}^{+},$$

where $ \mathcal{M}^{+}$ denotes the set of *P* × *P* symmetric positive definite matrices. Hereafter, we take $ \boldsymbol{\mu}=0$, without loss of generality.

We assume the precision matrix $ \mathbf{\Omega}$ to be sparse and place a shrinkage prior on its off-diagonal elements and an exponential prior on the diagonal elements. We use a continuous spike-and-slab prior whose formulation allows for the shrinkage of small elements to zero using a mixture of two Gaussian distributions which accounts for the presence or absence of edges,

(2) $$\displaystyle p\left(\mathbf{\Omega},\mathbf{\delta}|\tau,\rho \right)\propto \prod \limits_{i< j}\mathcal{N}\left(\omega_{ij}|0,\frac{\nu_{\delta_{ij}}^{2}}{\tau}\right)\prod \limits_{i}\,\mathrm{Exp}\,\left(\omega_{ii}|\frac{\lambda}{2}\right)\mathbb{1}\left(\mathbf{\Omega}\in \mathcal{M}^{+}\right)\prod \limits_{i< j}\mathrm{Bern}\left(\delta_{ij}|\rho \right)\hspace{0pt},\quad $$

where $ \nu_{0},\nu_{1}> 0$ are set to small and large values respectively ($ \nu_{0}\ll \nu_{1}$, see Section 4.2), *τ* is a scaling parameter and $ \lambda > 0$ controls the typical size of the diagonal entries. Wang (2015) suggests that inference is insensitive to the choice of *λ* since the data typically provide sufficient information for estimating the diagonal; we set *λ* = 2 in order to make the diagonal entries a priori equal to one on average. We also assume that each edge is a priori independently included or excluded by modeling the latent binary variable *δ_ij_* as Bernoulli-distributed with unknown probability *ρ*, and place conjugate hyperpriors on *ρ* and *τ*,

(3) $$\displaystyle \rho \sim \mathrm{Beta}(a_{\rho},b_{\rho}),$$

 

(4) $$\displaystyle \tau \sim \mathrm{Gamma}(a_{\tau},b_{\tau}),$$

where $ a_{\tau}=b_{\tau}=2$, and $ a_{\rho}=1,b_{\rho}=P$ to induce sparsity (Ročková and George 2014).

Alternative shrinkage approaches, such as the horseshoe prior (Li et al. 2019a), have been proposed for graphical modeling. An advantage of the spike-and-slab formulation is that it permits estimating posterior probabilities of inclusion (PPIs) for the edges which enable direct selection. To perform edge selection and thus estimation of the graph structure, a 0.5 threshold on the PPIs, resulting in the median probability model (Barbieri and Berger 2004), may be used. Alternatively, thresholds corresponding to Bayesian false discovery rates (FDR) can be directly estimated from the PPIs (Newton et al. 2004; Supplementary Material S1.4). Under a deterministic inference framework, PPIs are typically well separated (Carbonetto and Stephens 2012; Ročková and George 2014), and different thresholding rules usually produce minor differences in estimations of the graph structure.

We hereafter abbreviate model (1)–(4) as “GM” (for vanilla spike-and-slab graphical model). When setting *τ* = 1, we recover the model used by Li and McCormick (2019), which is itself based on the general formulation of Wang (2015). As discussed in Osborne et al. (2022), inferring the scaling factor *τ* from the data in continuous spike-and-slab prior specifications allows adaptive learning of the scales and improves edge selection.

### 3.2 A framework for leveraging node-level information

The GM model (1)–(4) assumes that all nodes in the graph are exchangeable a priori, yet certain nodes may be affected by exogenous factors which make them more likely to have connections. Let $ \boldsymbol{V}\in \mathbb{R}^{P\times Q}$ be a matrix of *Q* node-level auxiliary variables, that may be informative on the degree of the *P* nodes in the network, ie its rows $ \boldsymbol{v}_{i},\,i=1,\ldots,P$, correspond to “observations” (or annotations) on node *Y_i_*. As motivated in Sections 1 and 2, we introduce a top-level model hierarchy that lets the propensity of nodes to have high degrees be informed by the *Q* auxiliary variables via a probit regression on the probability of edge inclusion,

(5) $$\displaystyle \begin{array}{c}
\delta_{ij}|\rho_{ij}\sim \mathrm{Bern}(\rho_{ij}),\quad 1\leq i< j\leq P,\\
\rho_{ij}=\Phi \left(\zeta +\sum \limits_{q=1}^{Q}V_{iq}\beta_{q}+\sum \limits_{q=1}^{Q}V_{jq}\beta_{q}\right)\hspace{0pt},\end{array}$$

where $ \Phi (\cdot)$ is the standard normal cumulative distribution function and *ρ_ij_* is the edge-specific spike-and-slab probability. Specifically, the inclusion of edge (*i*, *j*) depends on the overall network sparsity, controlled by *ζ*, and on the influence of the annotations for nodes *Y_i_* and *Y_j_*. Hence, $ \beta_{q}\neq 0$ indicates an effect of variable *V_q_* on the degrees of the nodes in the graph (ie on their “propensity” to be hubs). Other link functions could be used in place of the probit link, although its use is computationally appealing due to the possibility to use a data-augmentation formulation that ensures analytical inference updates (Supplementary Material S1.1). We complete the specification by assuming

(6) $$\displaystyle \zeta \sim \mathcal{N}(n_{0},t_{0}^{2}),$$

and

(7) $$\displaystyle\begin{align*}
\beta_{q}|\sigma^{2}&\sim N(0,\sigma^{2}),\quad q=1,\ldots,Q,\\
\sigma^{-2}&\sim \mathrm{Gamma}(a_{\sigma},b_{\sigma}),\end{align*}

where $ a_{\sigma}=b_{\sigma}=2$, and *n*_0_ and $ t_{0}^{2}$ are chosen to induce sparsity by matching prior guesses on the expectation and standard deviation for the number of edges in the network (see Section 3.4). We abbreviate model (1)–(2), (4)–(7) as “GMN”, for spike-and-slab graphical model with normal prior for the node-level auxiliary variable effects.

### 3.3 A framework for selecting node-level information

The top-level regression framework (5)–(7) is well suited to settings with a handful of auxiliary variables. Often, however, a large number of candidate auxiliary variables may be available to the practitioner, with the belief that only a few variables (if any) are relevant to the graphical structure of primary interest. We accommodate settings where *Q* is large by equipping the model with a selection prior that permits leveraging only the variables inferred as being informative, discarding the remaining ones as irrelevant. Specifically, we place a spike-and-slab prior on the effects of variables $ V_{q},\,q=1,\ldots,Q$,

(8) $$\displaystyle\begin{align*}
\beta_{q}|\gamma_{q},\sigma^{2}& \sim \gamma_{q}\mathcal{N}(0,\sigma^{2})+(1-\gamma_{q})\delta (\beta_{q}),\quad q=1,\ldots,Q,\\
\gamma_{q}|o&\sim \mathrm{Bern}(o),\\
o&\sim \mathrm{Beta}(a_{o},b_{o}),\\
\sigma^{-2}&\sim \mathrm{Gamma}(a_{\sigma},b_{\sigma}),\end{align*}

where $ \delta (\cdot)$ is the Dirac delta distribution. The binary latent parameter *γ_q_* indicates whether the *q*th variable influences the hub propensity of nodes in the graph, and *o* is the shared probability for variables to be included. The hyperpriors on *o* and on the slab variance $ \sigma^{2}$ are conjugate, and we set $ a_{\sigma}=b_{\sigma}=2$ (as for the GMN model), and $ a_{o}=1,b_{o}=Q$ to induce sparsity on auxiliary variable effects. We refer to model (1)–(2), (4)–(6), (8) as “GMSS”, for graphical model with spike-and-slab prior for the node-level auxiliary variable coefficients.

As for the bottom-level model on the edges (1)–(2), the spike-and-slab prior formulation (8) for the auxiliary variables conveniently yields PPIs which allow directly pinpointing the relevant variables from a potentially large set of candidate auxiliary variables. Hence, thanks to this sparse selection, the GMSS model not only improves the estimation of edges by exploiting variables inferred as relevant to the dependence structures (and only those), but also permits a data-driven discovery of these auxiliary variables. Inspecting the selected variables may be particularly informative to generate hypotheses on the mechanisms underlying the uncovered network dependence structures.

In summary, inference for the GMSS model yields two sets of spike-and-slab PPIs which will be our main quantities of interest: PPIs based on the binary latent parameters $ \{\delta_{ij},\,1\leq i< j\leq P\}$ for the inclusion of edges, and PPIs based on the binary latent parameters $ \{\gamma_{q},\,1\leq q\leq Q\}$ for the inclusion of auxiliary variables.

**Fig. 1:**
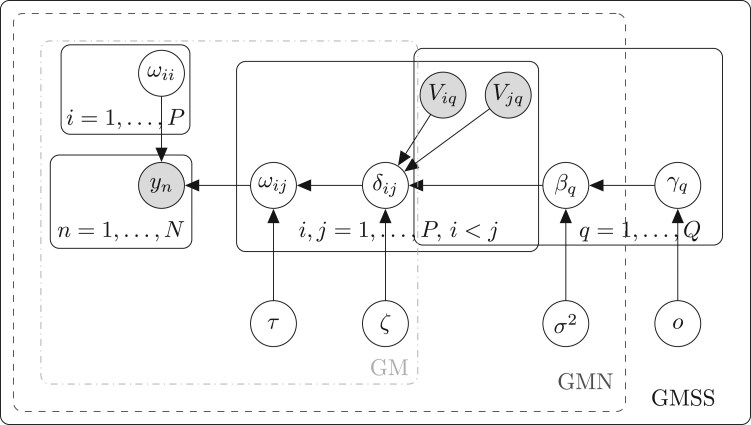
Graphical representation of the GMSS model (1)–(2), (4)–(6), (8). The shaded nodes are observed, and the others are inferred. The grey plates delineate the simpler models detailed in Sections 3.1 and 3.2, ie GM: light grey dashed-dotted plate; GMN: dark grey dashed plate.

The posterior distribution of the GMSS model is

(9) $$\displaystyle \begin{array}{c}
p(\mathbf{\Omega},\boldsymbol{\delta},\tau,\zeta,\boldsymbol{\beta},\boldsymbol{\gamma},o,\sigma^{2}|\boldsymbol{Y})\\
\quad \propto p\left(\boldsymbol{Y}|\mathbf{\Omega}\right)\,\mathbb{1}\left(\mathbf{\Omega}\in \mathcal{M}^{+}\right)\prod \limits_{i=1}^{P}p\left(\omega_{ii}\right)\prod \limits_{i< j}\left\{p\left(\omega_{ij}|\delta_{ij},\tau \right)p\left(\delta_{ij}|\zeta,\boldsymbol{\beta}\right)\right\}\\
\quad \quad \times p\left(\tau \right)p\left(\zeta \right)\prod \limits_{q=1}^{Q}\left\{p\left(\beta_{q}|\gamma_{q},\sigma^{2}\right)p\left(\gamma_{q}|o\right)\right\}p\left(o\right)p\left(\sigma^{2}\right),\end{array}$$

and its graphical representation, as well as those of the GM and GMN models, is provided in Fig. 1.

### 3.4 Hyperprior elicitation for the network sparsity

In this section, we detail our procedure to elicit the top-level hyperparameters for the prior $ \zeta ~ \sim ~ \mathcal{N}(n_{0},t_{0}^{2})$. While placing hyperpriors on *n*_0_ and $ t_{0}^{2}$ is in principle feasible, we instead choose to limit the model complexity using a hyperparameter elicitation strategy that facilitates interpretation and allows flexibly encoding prior knowledge about the network sparsity. To this end, we repurpose ideas of Bottolo and Richardson (2010) and Ruffieux et al. (2020) from the regression setting to the graphical setting. Specifically, we consider the simplifying assumption of a network without the influence of auxiliary variables, ie where the probit submodel on the edge spike-and-slab probability *δ_ij_* in (5) becomes $ \delta_{ij}|\zeta \sim \mathrm{Bern}\{\Phi (\zeta)\}$. Noting that the first two moments for the prior number of edges in the network are a function of *n*_0_ and $ t_{0}^{2}$, we then propose to specify an expectation and a standard deviation for the prior number of edges in the network and solve numerically the system of equations to obtain the corresponding values for *n*_0_ and $ t_{0}^{2}$. Specifying prior numbers of edges in the network has a more intuitive interpretation than directly specifying the top-level hyperparameters *n*_0_ and $ t_{0}^{2}$. Detailed derivations and sensitivity analyses are in Supplementary Material S1.2. These analyses suggest that inference is robust to the prior expectation of the edge numbers, when the sparse choices are made. The elicitation of the prior standard deviation is even less important, although its influence increases when the prior mean is specified to reflect a graph that is denser than the true graph.

## 4 Fast deterministic inference

### 4.1 Variational expectation conditional maximization algorithm

Although block Gibbs sampling has been introduced for spike-and-slab graphical models (Wang 2015), MCMC approaches are computationally expensive for high-dimensional graphs due to slow mixing. To address this, expectation conditional maximization (ECM) procedures have recently been put forward in the context of graphical models, as faster yet accurate deterministic alternatives to sampling-based approaches (Li and McCormick 2019; Gan et al. 2019; Deshpande et al. 2019; Bai et al. 2021). The ECM algorithm is a generalized expectation maximization (EM) algorithm, which replaces complicated maximization steps with several simpler conditional maximization steps (Meng and Rubin 1993, see Supplementary Material S1.1.1). Although computationally convenient, the ECM algorithm provides point estimates only, thus failing to quantify the uncertainty associated with parameter estimates. Here we propose a variational Bayes expectation conditional maximization (VBECM) algorithm, which couples conditional maximization (CM) updates for the precision matrix $ \mathbf{\Omega}$ with approximations of the full posterior distributions of all other model parameters, hereafter gathered in $ \mathbf{\Theta}=(\boldsymbol{\delta},\tau,\zeta,\boldsymbol{\beta},\boldsymbol{\gamma},o,\sigma^{2})$. Specifically, instead of finding point estimates, variational inference aims to estimate the posterior distribution (9) with an analytical approximate distribution $ q(\cdot)$ that minimizes the reverse Kullback-Leibler divergence with the true posterior,

$$\displaystyle \mathrm{KL}\left(q||p\right)=-\mathbb{E}_{q(\mathbf{\Omega},\mathbf{\Theta})}\, \log \,\left\{\frac{p(\mathbf{\Omega},\mathbf{\Theta}|\boldsymbol{Y})}{q(\mathbf{\Omega},\mathbf{\Theta})}\right\},$$

where $ \mathbb{E}_{q(\mathbf{\Omega},\mathbf{\Theta})}(\cdot)$ is the expectation with respect to the variational distribution $ q(\mathbf{\Omega},\mathbf{\Theta})$. This is equivalent to maximising the following a lower bound on the marginal log-likelihood,

(10) $$\displaystyle \mathcal{L}(q)=\mathbb{E}_{q(\mathbf{\Omega},\mathbf{\Theta})}\, \log \,p(\boldsymbol{Y},\mathbf{\Omega},\mathbf{\Theta})-\mathbb{E}_{q(\mathbf{\Omega},\mathbf{\Theta})}\, \log \,q(\mathbf{\Omega},\mathbf{\Theta}),$$

which is also known as the *evidence lower bound* (ELBO; Bishop and Nasrabadi 2006). Since the ELBO does not involve the expression of the marginal likelihood, it can conveniently be used as an objective function.

To find the optimal variational distribution, we rely on a *mean-field* approximation that assumes independence for a partition of the model parameters as follows,

(11) $$\displaystyle q\left(\mathbf{\Omega},\mathbf{\Theta}\right)=q\left(\mathbf{\Omega}\right)\prod \limits_{i< j}q\left(\delta_{ij}\right)q\left(\tau \right)q\left(\zeta \right)\prod \limits_{q=1}^{Q}q\left(\beta_{q},\gamma_{q}\right)q\left(o\right)q\left(\sigma^{2}\right).$$

This factorization is based on the following considerations. First, since $ \mathbf{\Omega}$ is positive definite, its entries cannot be treated independently. We thus model it using a multivariate variational factor. The spike-and-slab parameters for each auxiliary variable *V_q_*, $ q=1,\ldots,Q$, are also modeled jointly based on the factorization

(12) $$\displaystyle q(\beta_{q},\gamma_{q})=q(\beta_{q}|\gamma_{q})q(\gamma_{q}),$$

which retains the mixture structure and thus posterior multimodality. The remaining independence assumptions enable closed-form updates; their possible influence on inference will be assessed in simulations through evaluation of the edge and variable selection performance (Section 5.2).

We perform the optimization with a coordinate ascent algorithm that iteratively optimizes each factor of the mean-field approximation (11) while keeping the other factors fixed. The variational distribution for $ \mathbf{\Omega}$ is intractable, so we obtain a conditional maximization instead. Specifically, we reframe Wang (2015)’s block Gibbs sampling procedure as a CM step in an ECM procedure, which is equivalent to considering the variational distribution of $ \mathbf{\Omega}$ as being a point mass. The full derivations are provided in Supplementary Material S1.1.2.

### 4.2 Parallel grid search procedure for spike-and-slab variances

The GMSS model relies on two distinct spike-and-slab formulations: a continuous formulation for the edge effects, where the spike component is modeled using a peaked Gaussian distribution with variance $ \nu_{0}^{2}$ smaller than the slab variance $ \nu_{1}^{2}$, and a discrete formulation for the auxiliary variable effects, where the spike component is modeled using a Dirac delta point mass at zero. Continuous spike-and-slab priors have been used to investigate theoretical properties of mixture priors (George and McCulloch 1993; Ishwaran and Rao 2005; Narisetty and He 2014), but tend to be less commonly used than their discrete counterpart, partly because they require specifying two variance parameters ($ \nu_{0}^{2}$ and $ \nu_{1}^{2}$) instead of one only ($ \nu_{1}^{2}$) for discrete spike-and-slab priors. However the original spike-and-slab graphical model by Wang (2015) and its subsequent variants (e.g. Li and McCormick 2019; Li et al. 2019b) use the continuous spike-and-slab to model edge effects as this allows obtaining Gibbs samplers or ECM algorithms that maintain the positive definiteness constraint on the precision matrix at each iteration (provided a positive definite matrix is used to initialize $ \mathbf{\Omega}$). Here we rely on the same updating strategy (see Supplementary Material S1.1.2) and thus use a hybrid formulation with a continuous spike-and-slab prior to model edge effects and a discrete spike-and-slab prior to model auxiliary variables effects.

Appropriately eliciting the variances $ \nu_{0}^{2}$ and $ \nu_{1}^{2}$ in the continuous spike-and-slab formulation (2) is crucial as, along with the hyperparameter setting for *ζ* (Section 3.4), their values determine the level of regularization. In principle, both variances could be inferred simultaneously, however, this tends to yield degenerate solutions under empirical Bayes settings when the problem is sparse (Scott and Berger 2010; van de Wiel et al. 2019). To bypass this issue, proposals have been made to explore a series of spike variances while keeping the slab variance fixed (Ročková and George 2014, 2018; Li and McCormick 2019). The spike variance can be set based on a prior guess of the network sparsity (Osborne et al. 2022), cross-validation procedures (Li and McCormick 2019), or model selection criteria such as the Akaike information criterion (AIC; Akaike 1998; Langfelder and Horvath 2007), the Bayesian information criterion (BIC; Schwarz 1978; Jewson et al. 2022) and the extended Bayesian information criterion (EBIC; Chen and Chen 2008). Alternatively, it has been proposed to choose the spike variance by inspecting the stabilization of precision matrix estimates when conducting sequential runs of the algorithm whereby the variance is decreased progressively. For each run, parameters are initialized using the “optimal” values estimated at the previous run, thus resulting in a *dynamic posterior exploration* (Ročková and George 2018; Bai et al. 2021; Li et al. 2019a).

In practice, the sparsity is unknown and rough estimates are typically difficult to obtain without conducting full network inference, so selection based on sparsity is impractical. Cross-validation procedures have no guarantee for model selection consistency (Fan and Tang 2013) and are computationally prohibitive in realistic graphical settings. Dynamic posterior exploration has gained popularity in recent years, but guarantees of convergence to the optimal solution have yet to be established. In this paper, we propose a parallel grid-search procedure based on model selection criteria. Specifically, we set $ \nu_{1}=100$ and consider a series of candidate values for *ν*_0_ from grid ranging from $ 10^{-2}$ to 1. We then run the VBECM algorithm for the different choices of *ν*_0_ and select the value corresponding to the run with the lowest AIC. Our simulations show that the selected value usually corresponds to settings with the highest edge selection performance and good auxiliary variable selection performance and other model selection criteria produce similar results (Supplementary Material S1.3). Moreover, the selected *ν*_0_ is typically small, resulting in a small spike variance and thus small estimated precision matrix entries for edges inferred as “absent”. Importantly, the absence of sequential updates allows us to launch all the runs in parallel, which results in a highly efficient search. In particular, if the number of cores equals or exceeds the number of values in the grid, the runtime is dominated by the most computationally intensive run across the grid.

Our approach is implemented in the publicly available R package navigm (node-level auxiliary variables for improved graphical model inference).

## 5 Simulations

### 5.1 Data generation and simulation set-up

The numerical experiments presented below are meant to (i) assess the performance of our approach for estimating edges (adjacency matrix) and identifying “active” variables, ie node-level variables that are informative on the degrees or “centrality” of nodes, and therefore on the dependence structures in the network; (ii) benchmark it against state-of-the-art graphical modeling approaches on synthetic data designed to emulate real settings. We simulate networks with *N* samples and *P* nodes as follows. First, we generate *Q* candidate auxiliary variables from a right-skewed beta distribution with parameters 0.05 and 0.2, resulting in entries being mostly close to 0 with a few close to 1, and we gather them in a *P* × *Q* matrix ***V***. We further randomly select $ Q_{0}\leq Q$ active variables, and simulate their effects from a log-normal distribution with mean 0.5 and standard deviation 0.1; these effects are positive as we are particularly interested in emulating problems where features are associated with the presence of hubs, as motivated in Sections 1 and 2. Although negative effects of auxiliary variables may not be a primary concern in practice, our modeling framework can also detect such effects, as it enforces no restriction on the sign of effects. For completeness, we describe simulations in Supplementary Material S2.3 that comprise negative effects and a combination of positive and negative effects.

Unless stated otherwise, we choose *ζ* such that the network sparsity is $ \approx 3\% $. We then construct a binary adjacency matrix ***A*** for the graph skeleton: we establish its (*i*, *j*)th entry by thresholding the probability parameter *ρ_ij_* in (5) at 0.5 (median probability model), and set to 1 an additional percentage of zero entries in the upper triangular part of $ \boldsymbol{A}$ to include edges that are not induced by auxiliary variables (hereafter set to 10%, unless otherwise specified and referred to as “noise level”). Next, we generate the precision matrix $ \mathbf{\Omega}$ based on the structure of the adjacency matrix as follows (Tan et al. 2014),

$$\displaystyle\begin{align*}
E_{ij}&\sim \begin{cases}
0,& A_{ij}=0,\\
\mathrm{Unif}(-0.75,-0.25)\cup \mathrm{Unif}(0.25,0.75),& A_{ij}\neq 0,\end{cases}
\\
\overline{\boldsymbol{E}}&=\frac{1}{2}\left(\boldsymbol{E}+\boldsymbol{E}^{T}\right),\\
\mathbf{\Omega}&=\overline{\boldsymbol{E}}+\left(0.1-\lambda_{E}^{ \min }\right)\boldsymbol{I}_{P},\end{align*}

where $ \boldsymbol{I}_{P}$ refers to the *P* × *P* identity matrix, and $ \lambda_{E}^{ \min }$ represents the smallest eigenvalue of ***E***. This construction guarantees that the precision matrix $ \mathbf{\Omega}$ is symmetric positive definite and matches the structure of the adjacency matrix ***A***. Finally, we simulate *N* samples independently from the multivariate normal distribution $ \mathcal{N}_{P}(0,\mathbf{\Omega}^{-1})$. In each experiment, we generate 100 data replicates and summarize the performance across all the replicates. R code for reproducing all simulation results presented in the subsequent sections is available at https://github.com/XiaoyueXI/navigm_addendum.

### 5.2 Edge and auxiliary-variable selection performance

In this section, we evaluate the edge-selection performance of our modeling framework when encoding external information on the network structure, as well as its ability to identify the auxiliary variables relevant to this structure. We discuss two scenarios: a problem with a small number of auxiliary variables, where no selection is needed, and a problem with a large number of candidate variables of which only a subset is active, thus benefiting from sparse selection.

For the first scenario, we simulate data with *N* = 200 samples and *P* = 100 nodes whose hub structure is influenced by three variables, ie *Q* = 3 and $ Q_{0}=3$. We compare the performance of the vanilla GM model, which does not incorporate any auxiliary information, with that of the GMN model, which represents the effects of the auxiliary variables using a normal prior (see Fig. 1). To ensure comparability between the two models, we replace the GM model’s beta prior specification in (3) with a normal prior via a probit submodel, namely, $ \rho =\Phi (\zeta)$ (Supplementary Material S2.1); we hereafter refer to this modified model as “GM$^{*}$”.

**Fig. 2:**
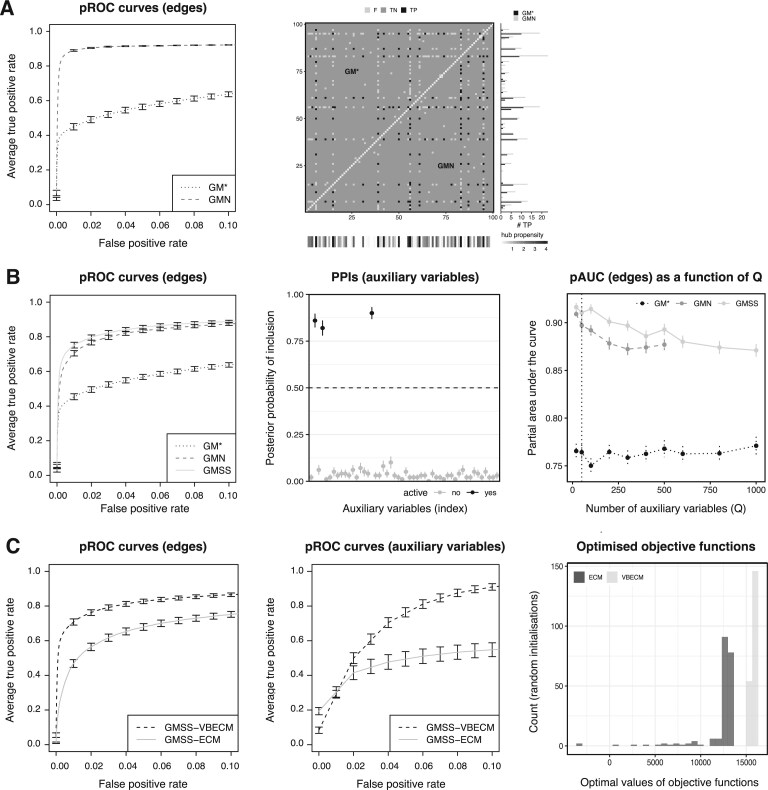
Performance for a problem with *N* = 200 samples, *P* = 100 nodes and 3 active auxiliary variables (100 replicates) under different scenarios. A) Problem with a small number of candidate auxiliary variables, ie *Q* = 3; B) problem with a large number of candidate auxiliary variables, ie *Q* = 50; C) comparison of VBECM and ECM inference for the GMSS model, on a problem with *Q* = 50 variables. (A-C, left) Average pROC curves, with standard error bars for edge selection. (A, middle) Heatmap of the graph recovery performance for the first replicate. Each entry corresponds to a pair of nodes (presence/absence of edges between them), with “F” (false positives or false negatives, light grey), “TN” (true negatives, dark grey) and “TP” (true positives, black), using GM$^{*}$ (top-left triangle) and GMN (bottom-right triangle). The contribution of auxiliary variables in influencing the node degrees (or “hub propensity”), ie $ \boldsymbol{v}_{\boldsymbol{i}}\mathbb{E}_{q(\mathbf{\Omega},\mathbf{\Theta})}(\boldsymbol{\beta})$ for node *i*, is shown in the bottom margin. The total number of true positive edges for each node is compared between GM$^{*}$ (black) and GMN (grey) in the right margin. (B, middle) Average PPIs of the candidate auxiliary variables, estimated with GMSS, with standard error bars. The variables simulated as active are in black, and the others in grey. (B, right) Average pAUC for edge-selection as a function of *Q*, along with standard errors. The line for GMN is truncated as the method could not converge within 1.5 day on an Intel Xeon CPU, 2.60 GHz machine. The vertical black dotted line represents the edge selection performance in the reference scenario. (C, middle) Average pROC curves for auxiliary variable selection using GMSS. (C, right) Optimal values of the objective function reached in 200 runs of the GMSS-ECM (*Q* function) and GMSS-VBECM (ELBO) algorithms using different random starts for the first replicate.

We assess performance using average partial receiver operating characteristic (pROC) curves, as follows: for each of 100 data replicates, we obtain a pROC curve by applying varying thresholds to the inferred posterior probabilities of inclusion (of edges or auxiliary variables), for false positive rates between 0 and 0.1. We then construct an average curve across replicates, with standard error bars. As expected, Fig. 2A indicates that the encoding of the three variables aids the recovery of edges in the network. Inspecting the reconstructed graph for a given replicate shows that inference using the GMN model results in a larger number of true positives compared to the GM$^{*}$ model. This example, where all candidate variables contribute to the node dependence structure, serves as an “easy” setting to check that the model indeed improves network inference when relevant auxiliary information is used.

We next consider the more challenging setting where a large number of candidate auxiliary variables, *Q* = 50, are measured, yet only sparsely related with the network structure, ie with zero effect for most variables, except for $ Q_{0}=3$ active variables. We refer to this setting as our “reference” data generation scenario. With its top-level spike-and-slab formulation, the GMSS approach accounts for the sparse nature of the auxiliary variable effects, while also providing intuitively appealing PPIs for each variable, which are unavailable from the GMN approach.

Figure 2B compares GMSS, GMN and GM$^{*}$, and again shows an improved edge selection performance when the auxiliary variables are leveraged, with GMSS and GMN (see also Supplementary Material S2.2). In addition, while the estimation of the *graph structure* is the primary focus of our work, the *precision matrix estimates* also indirectly benefit from the encoding of auxiliary data via the GMSS spike-and-slab binary latent variables. Indeed, the average mean absolute error of precision matrix estimates for true edges reported by either method reduces from 0.49 (s.e. 0.00) using GM$^{*}$ to 0.22 (s.e. 0.01) using GMSS. Figure 2B also shows a modest advantage for GMSS compared to GMN, likely due to the validity of sparsity assumption on the auxiliary variable effects and the accurate recovery of these effects. Indeed, the three variables relevant to the graph structure are correctly identified by GMSS: the average PPIs for these variables exceed 0.5, while those for the remaining variables, simulated as “inactive”, are below 0.25. From now on, our primary intent in discussing GMN is to contrast it with GMSS and underscore the possible the statistical and computational advantages of the latter method which is tailored to large *Q* data settings.

Table 1 generalizes these observations to different data generation scenarios, namely, for a grid of average sparsity and noise levels, and numbers of candidate auxiliary variables *Q*, active variables *Q*_0_, samples *N* and nodes *P*. It shows the average standardized partial areas under the curve (pAUCs) for the edge and auxiliary variable selection performance. As expected, irrespectively of the modeling approaches (GM$^{*}$, GMN, GMSS), edge estimation is more reliable when the network sizes are small (compare scenarios 1 with 2, and 3 with 4) and sample sizes are large (compare scenarios 1 with 3, and 2 with 4). Moreover, for given network sizes and sample sizes, estimation typically gets more challenging as (i) the graph gets denser (compare, e.g. scenarios 11 with 12), (ii) the number of active auxiliary variables gets larger (compare, e.g. scenarios 7 with 8), or (iii) the noise level (proportion of edges not influenced by auxiliary variables) gets larger (compare scenarios 1, 9 and 10).

**Table 1:** Performance for a grid of 12 data generation scenarios, using the GM$^{*}$, GMN, and GMSS models, showing the mean and standard error (in parentheses) of pAUCs for edge selection (for GM$^{*}$, GMN, and GMSS) and auxiliary variable selection (for GMSS) based on 100 data replicates.*^a^*

	Sparsity	Noise	*Q*_0_	*Q*	*N*	*P*	Edge selection		Variable selection	
							GM$^{*}$	GMN	GMSS	
1.						100	0.76 (0.01)	**0.90 (0.01)**	**0.91 (0.01)**	0.90 (0.01)
2.					200	50	0.89 (0.01)	**0.94 (0.00)**	**0.93 (0.01)**	0.84 (0.01)
3.					100	100	0.72 (0.01)	**0.84 (0.01)**	**0.83 (0.01)**	0.84 (0.01)
4.				50		50	0.83 (0.01)	**0.91 (0.01)**	**0.90 (0.01)**	0.80 (0.01)
5.				20			0.77 (0.01)	0.91 (0.00)	**0.92 (0.00)**	0.87 (0.01)
6.			3	100			0.75 (0.01)	0.89 (0.01)	**0.91 (0.00)**	0.92 (0.01)
7.			1				0.74 (0.01)	0.95 (0.00)	**0.96 (0.00)**	0.99 (0.00)
8.		10%	5				0.76 (0.01)	**0.89 (0.01)**	**0.88 (0.01)**	0.82 (0.01)
9.		20%					0.77 (0.01)	**0.88 (0.01)**	**0.88 (0.01)**	0.91 (0.01)
10.	3%	30%					0.77 (0.01)	0.84 (0.00)	**0.86 (0.00)**	0.90 (0.01)
11.	1%						0.87 (0.01)	**0.95 (0.00)**	0.94 (0.00)	0.85 (0.01)
12.	8.5%	10%	3	50	200	100	0.66 (0.00)	0.83 (0.01)	**0.89 (0.01)**	0.93 (0.01)

a Scenario 1 (first row) corresponds to the “reference” simulation presented in Fig. 2B, and shaded cells indicate the same settings as this scenario. For each scenario, the average pAUCs within one standard error of the highest average pAUC are highlighted in bold.

We also find that GMN and GMSS always outperform GM$^{*}$ in terms of edge selection. Moreover, as for the previous simulation example (Fig. 2B), the sparse selection of auxiliary variables induced by the top-level spike-and-slab submodel of GMSS results in comparable or improved graph recovery compared to GMN. Figure 2B also presents this comparison as a function of *Q*; it confirms a modest yet consistent outperformance of GMSS over GMN. Importantly, GMN becomes computationally intractable as *Q* increases, failing to converge within 1.5 days as *Q* > 500. In general, GMSS can save a factor of up to 3 in computational time compared to GMN (Supplementary Material Table S2), likely due to the validity of the sparsity assumption again: indeed the spike-and-slab prior (GMSS) permits a more efficient exploration of the posterior space compared to the “non-sparse slab-only” normal prior (GMN). Finally, Table 1 shows that the GMSS average pAUCs for the auxiliary variable selection based on the estimated PPIs range from 0.80 to 0.99, which suggests a very good recovery of the variables relevant to the graph structure; again, such PPIs constitute directly interpretable posterior quantities which confers a clear advantage to the GMSS approach when *Q* exceeds a handful of variables.

### 5.3 Null scenario and robustness to model misspecification

We next discuss two simulation scenarios which depart from data settings for which GMSS is primarily designed. We first consider a scenario where the graph dependence structure is not influenced by any auxiliary information, ie the top-level auxiliary variable model is a null model ($ Q_{0}=0$). Our goal is to evaluate the behavior of our approach when *Q* = 50 irrelevant variables are used as candidate auxiliary variables. GMSS correctly discards all variables as irrelevant to the graph structure, as their PPIs are all very low < 0.1. This suggests that large panels of candidate variables, whose relevance for the underlying graph is unclear, can be safely supplied to the GMSS approach: the spike-and-slab prior formulation not only allows pinpointing the relevant variables but also ensures that the irrelevant variables are correctly dismissed as “noise”. In contrast, GMN, which relies on a “slab-only” Gaussian prior, cannot handle a large number of (irrelevant) variables. It produces unstable inferences, with highly variable estimates across replicates (Supplementary Material S2.4). Such insufficient regularization of the auxiliary variable effects translates into a significantly lower edge selection performance (average pAUC: 0.55, s.e. < 0.01) compared to GMSS (average pAUC: 0.70, s.e. < 0.01). Our explorations further indicate that the GMN algorithm can be sensitive to parameter initializations, especially in weakly informative data settings.

We next evaluate the robustness of GMSS to model misspecification in a simulation setting where the *similarity between attributes of pairs of nodes* influences the presence or absence of edges between the nodes, but not the *values* of the node attributes (encoded as auxiliary variables) themselves. For instance, brain connectivity networks describe the connectivity between regions of interest (ROIs) within individual brains. They are typically modeled by Gaussian graphical models based on functional magnetic resonance imaging (fMRI) signals at each of the ROIs for a large number of images obtained during a scanning session (Higgins et al. 2018). The distance between these ROIs may influence their connectivity (Bu and Lederer 2021). We consider a simulation study assuming a misspecified edge regression model to mimic the aforementioned example. For each of 100 replicates, we simulate 50 variables of which 2 are relevant in the sense that edges are more likely to be present between nodes with similar values of these two variables. We generate datasets with *N* = 200 samples and *P* = 100 nodes under such a similarity-based edge model and use GMSS to estimate the graph and effects of auxiliary variables. In this setting, GMSS exhibits the desirable behavior of discarding all auxiliary variables as irrelevant (PPIs < 0.1) to the hub propensity of the nodes, akin to the null model scenario case (see Supplementary Material S2.5 for details).

### 5.4 Comparison with existing inference approaches

As motivated earlier, no graphical modeling approach exploiting auxiliary node-level variables exists, to our knowledge. It is nevertheless important to assess the accuracy and robustness of our VBECM algorithm by comparing it to other inference algorithms. To this end, we first consider the EMGS approach proposed by Li and McCormick (2019), which is based on a model similar to the GM model presented in Section 3.1 (see also a graphical representation of Fig. 1), with the following differences: (i) the GM model has added flexibility with a scale parameter *τ* in the edge-level spike-and-slab prior (fixed to 1 for EMGS), and (ii) the GM model parameters are inferred using variational inference (rather than pure ECM in EMGS).

Here we focus on evaluating the benefits of (ii), that is, of full posterior distribution estimation by variational inference. To this end, we compare our VBECM implementation for the GM model (hereafter referred to as “GM-VBECM”), with a pure ECM implementation of the same model (“GM-ECM”). We generate 100 datasets under the vanilla scenario where the graph structure does not depend on auxiliary information, for a problem with *N* = 200 samples and *P* = 100 nodes. The edge-selection performance of the GM-VBECM and GM-ECM runs is almost the same, based on the same hyperparameter and initialization choices to ensure fair comparisons. The computational time is comparable for the two inference algorithms, both of which take less than 2 seconds on average.

We next compare VBECM and ECM inference for the GMSS model, based on a problem where the graph structure is influenced by auxiliary variables. We use again the “reference” data generation scenario, with *N* = 200 samples, *P* = 100 nodes and *Q* = 50 auxiliary variables, of which $ Q_{0}=3$ contribute to the node degrees. Figure 2C shows the average pROC curves for edge selection and auxiliary variable selection. Here, the benefits of approximating the full posterior distributions with variational inference are clear. The improved performance is likely largely imputable to the ability of the VBECM algorithm to capture parameter uncertainty—notably thanks to the expressive joint variational approximation (12) for *β_q_* and *γ_q_*—and, as a result, to be less prone to entrapment in local modes, unlike the ECM algorithm. This is suggested by the large variability of optimal objective functions obtained from multiple restarts of the ECM algorithm applied to the first data replicate; in contrast, the VBECM algorithm consistently reaches similar values for the optimized ELBO (Fig. 2C and Supplementary Material S2.6). A comparison of ECM and VBECM for the effects of the auxiliary variables further show that the variational posterior means are more accurate than the ECM point estimates. Additionally, unlike GMSS-ECM, GMSS-VBECM produces credible intervals, and these cover the true value in most simulation replicates, despite the well-known tendency of variational inference to underestimate posterior variances (Supplementary Material S2.7).

Finally, unlike variational updates which can be obtained for model components with discrete point mass distributions, the ECM algorithm necessitates the use of a continuous spike-and-slab prior for the top-level auxiliary variable effects, as it requires taking derivatives of the objective function. This implies the use of an additional grid search for the spike variance, as described for the bottom-level edge effects (Section 4.2). This requirement may also partly contribute to the performance gap observed in Fig. 2C, and impacts computational efficiency substantially; the average runtime for the example of Fig. 2C is $ \approx 6$ minutes for GMSS-ECM and only 40 seconds for GMSS-VBECM.

### 5.5 Runtime profiling

**Fig. 3:**
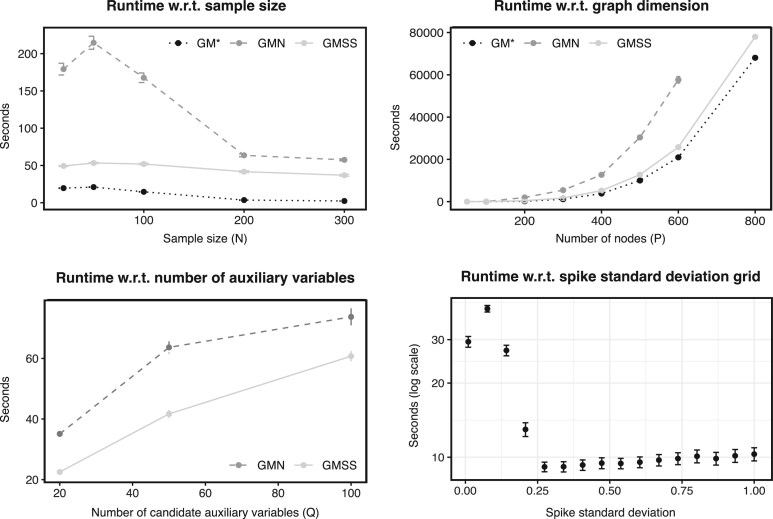
Runtime profiles in seconds on an Intel Xeon CPU, 2.60 GHz. Average time until convergence in seconds against sample sizes (top left), network sizes (top right) and the number of auxiliary variables (bottom left), with standard error bars based on 100 replicates. Bottom right: runtime breakdown for the “reference” scenario GMSS, with $ N=200,P=100,Q=50$, for a series of spike standard deviations on the *x*-axis. The runtimes reported in all the panels except the bottom right panel include the grid search procedure.

This section presents a systematic assessment of the computational efficiency of our framework. We again consider a grid of scenarios, based on variations of the “reference” scenario used in previous experiments. Specifically, Fig. 3 shows the runtimes obtained with GM$^{*}$, GMN, and GMSS when the sample sizes *N*, network sizes *P*, or number of auxiliary variables *Q* is varied. In all cases, the GMN approach requires the largest number of iterations to converge, making it the most computationally demanding approach, while the GM$^{*}$ approach is the fastest as it relies on a simpler model with no top-level hierarchy for auxiliary variables. All methods struggle to converge for very small sample sizes (although they all do converge, eventually) and the runtime is not increased as more information becomes available with larger sample sizes ($ N\leq 300$). However, because of the quadratic relationship between the number of nodes and the number of parameters, the runtime does increase substantially with the number of nodes, as well as with the number of auxiliary variables although to a lesser extent. A closer inspection of the “reference” scenario suggests that the large grid values for the spike standard deviation *ν*_0_ consume the least computational time. Indeed, in such cases, a larger number of negligible coefficients are absorbed into the spike distribution, resulting in sparser model, which, in turn, promotes fast convergence (see also, Wang 2015; Ročková and George 2014). All the computations are implemented on an Intel Xeon CPU, 2.60 GHz machine.

## 6 A study of gene regulation structure in monocyte

We return to the gene expression data presented in Section 2. Previous studies found substantial genetic regulation activity in monocytes on chromosome 12, likely linked with the development of immune-mediated diseases (Fairfax et al. 2012; Ruffieux et al. 2020). In particular, they identified a series of candidate “hotspots” (ie genetic variants controlling many gene levels) within 12 megabase pairs of the lysozyme gene *LYZ* (hereafter *LYZ* region), which is known to have an important role in human innate immunity (Fairfax et al. 2012; Kim et al. 2014; Ragland and Criss 2017). Here we undertake to clarify the mechanisms involved in the regulation activity of the *LYZ* region by estimating the network dependence structure of genes controlled by the hotspots.

As motivated in Section 2, we aim to use our GMSS approach to estimate the partial correlation structures of genes in monocytes from the expression data described in Fairfax et al. (2014), taking advantage of gene-level auxiliary variables on the genetic regulation of these genes. Specifically, to construct the auxiliary variables, we first analyze independent genetic and monocyte expression data from the CEDAR cohort (Momozawa et al. 2018) with the Bayesian joint mapping approach *atlasqtl* (Ruffieux et al. 2020) and obtain posterior probabilities of association between each pair of genetic variant and gene. We next filter the genetic variants and genes, retaining the 29 genetic variants, from the *LYZ* region, associated with at least one gene, and the 137 genes associated with at least one genetic variant from the *LYZ* region, using a permutation-based Bayesian FDR of 20% (see Ruffieux et al. 2020), for details. Finally, we define the auxiliary variables as the *P* × *Q* matrix ***V*** of pairwise posterior probabilities of association between the *P* = 137 genes and *Q* = 29 genetic variants. Note that this matrix is “weakly sparse” (most of its entries are close to zero), as most of the retained genetic variants are associated with one gene, and very few are large hotspots, associated with about half of the genes.

We apply GMSS to two monocyte expression datasets from the Fairfax et al. (2014) study, filtering the corresponding *P* = 137 genes. As introduced in Section 2, the first dataset involves genes quantified from unstimulated monocytes, for *N* = 413 subjects, and the second dataset involves genes quantified from monocytes that underwent stimulation by IFN-*γ* inflammatory proxies, for *N* = 366 subjects. To evaluate the benefits of using the genetic association information in $ \boldsymbol{V}$ with GMSS, we also compare our findings with those obtained from a classical spike-and-slab graphical model with no encoding of auxiliary variables, namely, using the GM$^{*}$ model. We do not consider GMN in this study for the statistical and computational reasons discussed in our simulations (Sections 5.2, 5.3 & 5.5); in particular, (i) unlike GMSS, it doesn’t entail a *selection* of auxiliary variables, which could shed light on biological mechanisms, (ii) its edge-selection performance tends to be lower than that of GMSS, for a longer runtime, and (iii) it can lead to unstable inference due to insufficient regularization.

The network sparsity estimated by the two approaches is similar, 3.6% (GM$^{*}$) and 3.9% (GMSS) for the unstimulated monocyte data, 4.1% (GM$^{*}$) and 4.3% (GMSS) for the stimulated monocyte data. The denser graphs in the latter case reflect previous findings showing that immune stimulation triggers substantial genetic activity, hence resulting in stronger gene dependence in the controlled networks. In the unstimulated monocyte networks, a total of 312 edges are shared by the graphs inferred by the two approaches, 25 edges are unique to the GM$^{*}$ graph, and 48 are unique to the GMSS graph. Similarly, in the stimulated monocyte networks, 346 edges are shared by the graphs inferred by the two approaches, 33 edges are unique to the GM$^{*}$ graph, and 56 are unique to the GMSS graph.

**Fig. 4:**
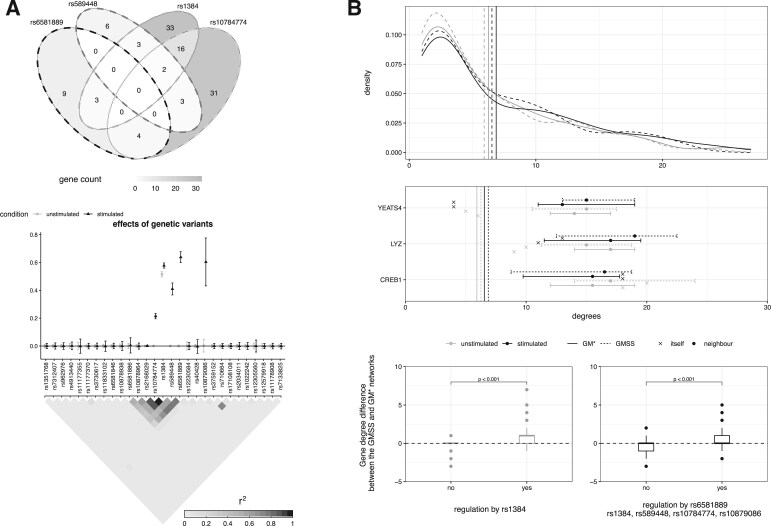
Monocyte networks estimated by GM$^{*}$ and GMSS. A) Top: number of genes associated with the four hotspot genetic variants picked by the GMSS in the stimulated monocyte network analysis in CEDAR dataset. Bottom: Estimated effects of auxiliary variables corresponding to genetic variants in the *LYZ* region and their *r* ^2^ measure of linkage disequilibrium (genetic correlation), using GMSS in the unstimulated (grey) and simulated (black) monocyte network analyses along with their 95% variational credible intervals. B) Top: degree distributions of gene networks inferred by GM$^{*}$ (solid lines) and GMSS (dash lines), when monocytes are unstimulated (grey) and stimulated (black). Average degrees are indicated by vertical lines. B) Middle: degrees of *LYZ*, *YEATS4* and *CREB1* using GM$^{*}$ (solid lines) and GMSS (dash lines), and median degree and interquartile range for their first-order neighbors. Bottom: difference in node degrees between the graphs inferred with GMSS and GM$^{*}$, for the gene sets categorized by the associations with genetic hotspot, rs1384, corresponding to the selected variable in the stimulated monocyte analysis, and genetic variants rs10879086, rs6581889, rs1384, rs589448, rs10784774, corresponding to the selected variables in the stimulated monocyte analysis. The *P*-values obtained from median-based permutation tests are shown. Genetic variants corresponding to the variables used as auxiliary data.

Inspecting the auxiliary variable PPIs estimated by GMSS, when monocytes are not stimulated, indicates that the only variable retained is that corresponding to the genetic variant rs1384 (using the median probability model rule PPIs > 0.5). Interestingly, this variant was the second largest hotspot in the *atlasqtl* analysis, where it was found to be associated with 57 genes at FDR 20%; while a few of these genes are on the same chromosome as rs1384, most genes are spread across the entire genome. Moreover, one typically expects that any given block of highly correlated genetic variants (“locus”) will entail one or a few independent “causal” variants (if any), affecting disease traits (see, e.g. Schaid et al. 2018). It is therefore not surprising that GMSS selects only one auxiliary variable, corresponding to a single hotspot genetic variant. In the stimulated monocyte analysis, however, GMSS selects five auxiliary variables. These include the variable corresponding to the hotspot rs1384, which is again found relevant to the graph structure. The remaining four variables correspond to genetic variants rs589448, rs6581889, rs10784774 and rs10879086, of which the first three are also hotspots, regulating 14, 16 and 56 genes, respectively. Compared to the unstimulated setting, this larger number of selected variables may again reflect the greater genetic activity expected in response to the stimulation intervention. The estimated effects of the auxiliary variables are reported in Fig. 4A (bottom). None of the variational credible intervals for the effects of the selected variables cover zero. Moreover, the variables corresponding to four hotspot genetic variants have smaller intervals, reflecting a higher level of certainty on their inclusion. Finally, this figure also depicts the correlation structure, or *linkage disequilibrium*, in the *LYZ* region for the genetic variants corresponding to the auxiliary variables in $ \boldsymbol{V}$. While rs10879086 displays very weak correlation with the other variants in the region, genetic variants rs10784774, rs1384, rs589448 and rs6581889 are in the same linkage disequilibrium block. However, they tend to control different sets of genes, as shown in Fig. 4A (top).

To understand the effect of accounting for the genetic regulation information $ \boldsymbol{V}$, we compare the degrees of the genes in the GMSS network with those in the GM$^{*}$ network which does not make use of this information. Figure 4B indicates that the degree distributions inferred from GM$^{*}$ and GMSS are similar, regardless of the stimulation condition. However Fig. 4B further shows that, in the unstimulated monocyte networks, the median degree of the set of genes regulated by the hotspot rs1384 is higher in the GMSS estimation compared to the GM$^{*}$ estimation, and the increment is significant compared to random sets of unregulated genes of the same size using permutation testing (empirical *P*-value $ < 10^{-3}$). A similar observation holds in the stimulated monocyte networks, for the set of genes regulated by the five genetic variants corresponding to the auxiliary variables selected by GMSS. This suggests that the genetic regulation information is relevant to the gene dependence structure and that the genetic variants influencing this structure are effectively singled out and leveraged thanks to the GMSS top-level spike-and-slab submodel on the auxiliary variable effects.

The previous observations also suggest that inspecting genes with high degrees (“hubs”) may be particularly helpful to understand the mechanisms by which genes regulated by genetic hotspots influence immune-mediated diseases. The lists of genes with the top 10% node degrees in the GMSS and GM$^{*}$ networks largely overlap, with 13 hub genes common to both methods in the unstimulated monocyte networks, and 14 in the stimulated monocyte networks (Supplementary Table S3). The tripartite motif containing 16 like pseudogene (*TRIM16L*) is the most central node in the unstimulated monocyte networks. Its degree is increased by 3 when auxiliary variables are exploited with the GMSS model (degree 27), compared to the GM$^{*}$ model (degree 24). The tripartite motif (TRIM) is a large human protein family which has been shown to have a role in innate immune signaling pathways (Carthagena et al. 2009; Yang et al. 2020). Although this motif still also appears among the top hubs in the stimulated monocyte networks, it has a lower degree (20 using both GM$^{*}$ and GMSS approaches), which aligns with the findings of Rajsbaum et al. (2008), reporting a down-regulation of TRIM16 in human macrophages stimulated with IFN-*γ*. The top hub in the stimulated monocyte networks is the tumor suppressor p53-binding protein 2 (*TP53BP2*). Its degree is increased by 4 in the GMSS network analysis (26) compared to the GM$^{*}$ analysis (22). Turnquist et al. (2014) shows that IFN-*γ* induces the expression of *TP53BP2* (also called *ASPP2*) in THP-1 cells, which are a type of human monocytic cell lines. Xie et al. (2022) found the deficiency of *TP53BP2* causes dysregulation of both adaptive and innate immune systems, yet further research is required to fully understand its role in immune regulation. These two examples suggest that GMSS likely helps pinpoint hub genes from important gene pathways, although their exact functions should be investigated further, for instance with experimental studies. Exploring the biological functions of other hubs may yield further valuable insights on the biological pathways implicated in diseases related to the innate immune system.

Finally, previous evidence suggests that hotspots from the *LYZ* region regulate genes located nearby, which themselves mediate the expression of remote genes (possibly on other chromosomes). Specifically, the *LYZ* gene itself and the YEATS domain containing 4 (*YEATS4*) gene, both located within the *LYZ* region, are candidate mediators thought to feed into the remote gene CAMP responsive element binding protein 1 (*CREB1*), located on chromosome 2 (Fairfax et al. 2012). Ruffieux et al. (2021) further hypothesized that *CREB1* may feed back onto *LYZ*. Interestingly these genes, and their first-order neighbors, also have peculiar hub patterns in our analyses (Fig. 4B). Both *LYZ* and *CREB1* exhibit larger degrees than the mean network degree, in the unstimulated and stimulated monocyte estimations obtained with both GMSS and GM$^{*}$ (degrees ranging from 9 to 20). In contrast, *YEATS4* has a relatively small degree (ranging from 5 to 7 depending on the method and stimulation condition), which may indicate that *LYZ* and *CREB1* are more promising candidate mediators than *YEATS4*, aligning with the hypothesis of Fairfax et al. (2012). Furthermore, *LYZ* and *CREB1* are direct neighbors in both the GM$^{*}$ and GMSS networks (Supplementary Material Tables S4 and S5). These observations lend additional support to the proposed hotspot-mediating role of *LYZ* and *CREB1*, and warrant further investigation about their mechanisms of action using experimental studies and independent data.

## 7 Discussion

We have introduced a novel framework for estimating sparse dependence structures in Gaussian graphical models by leveraging external auxiliary information on the network structure. Our approach (i) uses a top-level spike-and-slab formulation to infer and encode the effects of node-level candidate variables; (ii) implements a VBECM algorithm that outperforms existing ECM algorithms for graphical models while maintaining computational efficiency. Our simulations and monocyte gene expression study suggest a substantial gain in accuracy in estimating graphical structures when auxiliary variables are exploited using our framework. Importantly, we have shown that our approach permits hypothesis-free selection of relevant variables, effectively discarding all variables irrelevant to the estimation of the underlying network. This permits supplying large panels of variables, with tens or possibly hundreds of candidate auxiliary variables, in an agnostic fashion, without risk of worsening inference. Moreover, the spike-and-slab PPIs are interpretable quantities for selecting edges, as well as auxiliary variables which may be relevant to the graph structure. Hence, our approach not only enhances statistical power to detect weak edges thanks to the auxiliary variables, but also pinpoints which variables are likely informative, thereby offering insights for interpreting the uncovered edges and formulating plausible mechanistic hypotheses in applied settings. Finally, we have shown that our VBECM algorithm, which infers full posterior distributions, improves upon the current deterministic ECM algorithms used for fast network inference, without giving up on scalability.

To conclude, we would like to emphasize the versatility of our framework, whose applicability and usefulness range across different problem settings and areas. First, as outlined in Section 2, other gene-level variables may aid in reconstructing gene expression networks. In particular, gene memberships to biological pathways can be encoded as binary node-level variables. This may not only improve the detection of gene structures—since genes in the same pathway tend to gather as modules in gene networks (Langfelder and Horvath 2007)—but also allows automatically identifying “active” pathways relevant to the network, which may point toward specific disease processes underlying the gene dependence structures. Clearly, our approach is not limited to the study of gene networks but is well suited to any molecular entities whose levels can be modeled using a multivariate Gaussian distribution; this includes, for instance, protein-protein interaction networks (Wang et al. 2016) or metabolite-metabolite association networks (Cak ir et al. 2009). Our framework also has wide applications outside molecular research; to name only a few, it can be used to estimate (i) functional connectivity between brain regions (Belilovsky et al. 2016; Li and Solea 2018), for which features such as the size and brain lobe can serve as candidate auxiliary variables, (ii) relationships between diverse physical and mental health symptoms (Lafit et al. 2019), encoding the mapping of the different symptoms with different diseases as auxiliary variables, (iii) historical social networks, such as collaboration networks (Newman 2001; Li et al. 2020) or contact networks (Liljeros et al. 2001; Yang et al. 2021), whose estimation may be improved by leveraging the characteristics of individuals, (iv) movement networks such as networks of road traffic states (Hara et al. 2018), along with auxiliary information on road conditions and properties, (v) financial networks representing stock prices within banking systems (Nicola et al. 2020), where factors like bank ownership structures and sizes may enhance the detection of network edges.

Different methodological extensions can be considered. First, a natural development would be to adapt the framework to joint inference across multiple related networks. In such a setting, one could envision having network-specific auxiliary variable effects, or even network-specific sets of auxiliary variables. For instance, the monocyte study could benefit from analyzing genes collected from different cell types or tissues (“conditions”), and the auxiliary variables could be constructed from genetic regulation information in each of the conditions concerned. Since it is expected that genetic regulation and gene relationships are partly shared across several conditions, estimation would benefit from exploiting common information across networks. Such an extension could borrow ideas from existing proposals on Bayesian joint network inference, e.g. by Peterson et al. (2015), Li et al. (2019b) or Lingjærde et al. (2024), re-purposing them to the auxiliary variable setting. Furthermore, our modeling framework assumes a global influence of the selected auxiliary variables on the network structure. Modeling “local effects” on subsets of nodes may be more realistic in some applied settings. A careful extension of the model hierarchy, possibly considering domain-specific knowledge, may prove useful to capture nuanced relationships within complex networks. In addition, our modeling framework can be easily adapted to incorporate edge-level, rather than node-level information through the probit submodel on the probability of edge inclusion. Finally, it would be interesting to consider modeling variants based on alternative sparse prior distributions for edges and/or auxiliary variables, such as the horseshoe prior (Li et al. 2019a), or the spike-and-slab lasso which is based on a mixture of double exponential distributions combines ideas of penalized regression and spike-and-slab shrinkage (Ročková and George 2018; Bai et al. 2021).

Our framework serves as groundwork for more accurate, interpretable, versatile and scalable network estimation. We hope that it can pave the way to a more principled use of auxiliary variables in applied problems involving network inference, capitalizing on the wealth of annotation sources that are now available to practitioners.

## Supplementary Material

kxae021_Supplementary_Data

## Data Availability

The CD14$^{+}$ monocyte gene expression data presented in Fairfax et al. (2014) have been generated using HumanHT-12v4 arrays and are freely available from ArrayExpress (accession E-MTAB-2232). The CEDAR dataset (Momozawa et al. 2018) involves gene expression data from CD14$^{+}$ monocytes generated using Illumina HumanHT-12 v4 arrays and genotyping data generated using Illumina HumanOmniExpress-12 v1 A arrays. The raw expression data and genotype data are available from ArrayExpress (accession E-MTAB-6667 and E-MTAB-6666, respectively).

## Software and reproducibility

Our R package navigm (node-level auxiliary variables for improved graphical model inference) is available at https://github.com/XiaoyueXI/navigm. Replication code for all numerical examples presented in this article is available at https://github.com/XiaoyueXI/navigm_addendum.
