# Supplementary material for: A modeling framework for detecting and leveraging node-level information in Bayesian network inference
Source: Biostatistics. 2024 Jun 25;26(1):kxae021. doi: 10.1093/biostatistics/kxae021 (PMC11823055; doi:10.1093/biostatistics/kxae021)
Supplement: kxae021_Supplementary_Data [file kxae021_supplementary_data.pdf]

Supplementary material to "A modelling framework for detecting and leveraging node-level information in Bayesian network inference"

Xiaoyue Xi,
Hélène Ruffieux
MRC Biostatistics Unit, University of Cambridge

Contents

1 Fast deterministic inference **2**
1.1 Algorithms . . . 2
1.1.1 Derivation of the expectation conditional maximisation algorithm . . . 2
1.1.2 Derivation of the variational expectation conditional maximisation algorithm 5
1.2 Hyperparameter settings . . . 10
1.3 Parallel grid search procedure for spike-and-slab variances . . . 12
1.4 Bayesian false discovery rate . . . 13

2 Addendum to the simulation experiments **15**
2.1 Variants of the model . . . 15
2.2 Edge-selection performance . . . 15
2.3 Non-positive effects . . . 16
2.4 Null scenario . . . 16
2.5 A misspecified scenario: similarity-based edge effects . . . 17
2.6 Handling posterior multimodality . . . 18
2.7 Variational credible intervals . . . 19
2.8 Runtime profiling . . . 20

3 Addendum to the monocyte network application **22**

1 Fast deterministic inference

1.1 Algorithms

In this section, we first adapt the expectation conditional maximisation (ECM) algorithm of Li and McCormick (2019) to our modelling framework and then present our variational Bayes expectation conditional maximisation (VBECM) algorithm for inference.

Recall the full model: for N centred measurements, $\boldsymbol{Y} = (\boldsymbol{y_1}, \dots, \boldsymbol{y_N})$, and Q auxiliary variables, $\boldsymbol{V} = \left(V_1^T, \dots, V_Q^T\right)^T$ for P nodes in a graph,

$$
\begin{aligned}
\boldsymbol{y_n} &\overset{\text{iid}}{\sim} \mathcal{N}_P(\mathbf{0}, \boldsymbol{\Omega}^{-1}), \quad \boldsymbol{\Omega} \in \mathcal{M}^+, \quad n = 1, \dots, N, \\
\omega_{ii} &\sim \text{Exp}\,(\lambda/2)\,, \quad i = 1, \dots, P, \\
\omega_{ij} \mid \delta_{ij}, \tau &\sim \delta_{ij}\mathcal{N}\left(0, \nu_1^2/\tau\right) + (1 - \delta_{ij})\mathcal{N}\left(0, \nu_0^2/\tau\right), \quad \nu_0 \ll \nu_1, \quad 1 \le i < j \le P, \\
\delta_{ij} \mid \rho_{ij} &\sim \text{Bern}(\rho_{ij}), \quad \rho_{ij} = \Phi(\alpha_{ij}), \quad \alpha_{ij} = \zeta + \boldsymbol{v_i}\boldsymbol{\beta} + \boldsymbol{v_j}\boldsymbol{\beta}, \\
\beta_q \mid \gamma_q, \sigma^2 &\sim \gamma_q\mathcal{N}(0, \sigma^2) + (1 - \gamma_q)\delta(\beta_q), \quad \gamma_q \mid o \sim \text{Bern}(o), \quad q = 1, \dots, Q, \\
\zeta &\sim \mathcal{N}(n_0, t_0^2), \quad o \sim \text{Beta}(a_o, b_o), \quad \sigma^{-2} \sim \text{Gamma}(a_\sigma, b_\sigma),
\end{aligned} \tag{1}
$$

where $\mathcal{M}^+$ is the set of symmetric positive definite matrices, $\Phi(\cdot)$ is the standard normal cumulative distribution function and $\delta(\cdot)$ is the Dirac distribution. To obtain analytical updates, we reparameterise the probit link formulation (1) using the classical data-augmentation which introduces a latent vector $\boldsymbol{z}$ as follows

$$
\delta_{ij} \mid z_{ij} = \mathbb{1}\{z_{ij} > 0\}, \quad z_{ij} \mid \zeta, \boldsymbol{\beta} \sim \mathcal{N}\left(\alpha_{ij}, 1\right), \quad \alpha_{ij} = \zeta + \boldsymbol{v_i}\boldsymbol{\beta} + \boldsymbol{v_j}\boldsymbol{\beta}.
$$

1.1.1 Derivation of the expectation conditional maximisation algorithm

The ECM algorithm, initially proposed by Meng and Rubin (1993), introduces several simpler conditional maximisation steps to replace a complicated maximisation step for which no analytical form can be obtained. Li and McCormick (2019) were the first to use the ECM algorithm for the spike-and-slab graphical model. They employed a continuous spike-and-slab prior to ensure closed-form updates, which is achieved by repurposing the Gibbs sampling slicing procedure proposed by Wang (2015).

To obtain an ECM implementation of our approach, we replace the discrete spike-and-slab for the node-level auxiliary variable selection in (8) of the main text,

$$
\beta_q \mid \gamma_q, \sigma^2 \sim \gamma_q\mathcal{N}(0, \sigma^2) + (1 - \gamma_q)\delta(\beta_q), \quad \sigma^{-2} \sim \text{Gamma}(a_\sigma, b_\sigma),
$$

with a continuous formulation, as in (2),

$$
\beta_q \mid \gamma_q, \tau_2 \sim \gamma_q\mathcal{N}\left(0, \sigma_1^2/\tau_2\right) + (1 - \gamma_q)\mathcal{N}\left(0, \sigma_0^2/\tau_2\right), \quad \tau_2 \sim \text{Gamma}(a_\sigma, b_\sigma), \tag{2}
$$

for $q = 1, \dots, Q$, where $\sigma_0, \sigma_1 > 0$ are set to small and large values respectively (see Section 4.2 in the main text) and and τ_2 is a scaling parameter. In what follows we also denote by τ_1, the scale parameter τ in the bottom-level continuous spike-and-slab formulation for the edge selection in (2) of the main text.

We introduce the notation $\boldsymbol{\Theta} = (\boldsymbol{\Theta_1}, \boldsymbol{\Theta_2})$, with $\boldsymbol{\Theta_1} = (\tau_1, \tau_2, \zeta, \boldsymbol{\beta}, o)$ representing unknown parameters and $\boldsymbol{\Theta_2} = (\boldsymbol{\delta}, \boldsymbol{z}, \boldsymbol{\gamma})$ representing latent variables. Given the estimates from the previous iteration t, the objective function to optimise is

$$
\begin{aligned}
Q(\boldsymbol{\Omega}, \boldsymbol{\Theta_1} \mid \boldsymbol{\Omega}^{(t)}, \boldsymbol{\Theta_1}^{(t)}) &= \mathbb{E}_{\boldsymbol{\Theta_2}|\boldsymbol{\Omega}^{(t)},\boldsymbol{\Theta_1}^{(t)},\boldsymbol{Y}} \left\{ \log p(\boldsymbol{\Omega}, \boldsymbol{\Theta_1}, \boldsymbol{\Theta_2} \mid \boldsymbol{Y}) \mid \boldsymbol{\Omega}^{(t)}, \boldsymbol{\Theta_1}^{(t)}, \boldsymbol{Y} \right\} \\
&= \frac{N}{2}\log(|\boldsymbol{\Omega}|) - \frac{1}{2}\mathrm{tr}(\boldsymbol{Y}^{\boldsymbol{T}}\boldsymbol{Y}\boldsymbol{\Omega}) - \frac{\lambda}{2}\sum_{i=1}^{P}\omega_{ii} \\
&\quad - \log(\nu_1)\sum_{i<j}\mathbb{E}_{\cdot|\cdot}(\delta_{ij}) - \log(\nu_0)\sum_{i<j}\left\{1 - \mathbb{E}_{\cdot|\cdot}(\delta_{ij})\right\} + \frac{P(P-1)}{4}\log(\tau_1) \\
&\quad - \frac{\tau_1}{2}\sum_{i<j}\omega_{ij}^2\mathbb{E}_{\cdot|\cdot}\left(\frac{\delta_{ij}}{\nu_1^2} + \frac{1-\delta_{ij}}{\nu_0^2}\right) + (a_\tau - 1)\log(\tau_1) - b_\tau\tau_1 \\
&\quad + \sum_{i<j}\mathbb{E}_{\cdot|\cdot}\Big[\delta_{ij}\log\mathbb{1}\{z_{ij} > 0\} + (1-\delta_{ij})\log\mathbb{1}\{z_{ij} \le 0\}\Big] \\
&\quad - \frac{1}{2}\sum_{i<j}\left\{\mathbb{E}(z_{ij}^2) - 2\alpha_{ij}\mathbb{E}(z_{ij}) + \alpha_{ij}^2\right\} - \frac{\zeta^2}{2t_0^2} + \frac{n_0\zeta}{t_0^2} \\
&\quad - \log(\sigma_1)\sum_{q=1}^{Q}\mathbb{E}_{\cdot|\cdot}(\gamma_q) - \log(\sigma_0)\sum_{q=1}^{Q}\left\{1 - \mathbb{E}_{\cdot|\cdot}(\gamma_q)\right\} + \frac{Q}{2}\log(\tau_2) \\
&\quad - \frac{\tau_2}{2}\sum_{q=1}^{Q}\beta_q^2\mathbb{E}_{\cdot|\cdot}\left(\frac{\gamma_q}{\sigma_1^2} + \frac{1-\gamma_q}{\sigma_0^2}\right) + (a_\sigma - 1)\log(\tau_2) - b_\sigma\tau_2 \\
&\quad + \log(o)\sum_{q=1}^{Q}\mathbb{E}_{\cdot|\cdot}(\gamma_q) + \log(1-o)\sum_{q=1}^{Q}\left\{1 - \mathbb{E}_{\cdot|\cdot}(\gamma_q)\right\} \\
&\quad + (a_o - 1)\log o + (b_o - 1)\log(1-o) + \mathrm{cst},
\end{aligned}
$$

where $\mathbb{E}_{\cdot|\cdot}(\cdot) := \mathbb{E}_{\boldsymbol{\Theta_2}|\boldsymbol{\Omega}^{(t)},\boldsymbol{\Theta_1}^{(t)},\boldsymbol{Y}}(\cdot)$ denotes the expectation of the posterior conditional on current estimates and observations and cst is constant with respect to $\boldsymbol{\Omega}$ and $\boldsymbol{\Theta_1}$. The ECM algorithm iterates between an "expectation step" (E-step), which evaluates the conditional expectations in the objective function, and a "conditional maximisation step" (CM-step), which solves for mode of the objective function in a coordinate ascent manner, until the objective function converges. These steps are detailed below.

The E-step The conditional expectations in the objective function are

$$
\begin{aligned}
\mathbb{E}_{\cdot|\cdot}(\delta_{ij}) &= \frac{p\left(\omega_{ij}^{(t)} \mid \delta_{ij} = 1\right)\Phi\left(\alpha_{ij}^{(t)}\right)}{p\left(\omega_{ij}^{(t)} \mid \delta_{ij} = 1\right)\Phi\left(\alpha_{ij}^{(t)}\right) + p\left(\omega_{ij}^{(t)} \mid \delta_{ij} = 0\right)\left\{1 - \Phi\left(\alpha_{ij}^{(t)}\right)\right\}}, \\
\mathbb{E}_{\cdot|\cdot}(\gamma_q) &= \frac{p\left(\beta_q^{(t)} \mid \gamma_q = 1\right)o^{(t)}}{p\left(\beta_q^{(t)} \mid \gamma_q = 1\right)o^{(t)} + p\left(\beta_q^{(t)} \mid \gamma_q = 0\right)(1 - o^{(t)})},
\end{aligned}
$$

and

$$\mathbb{E}_{\cdot|\cdot}\left(\frac{\delta_{ij}}{\nu_1^2}+\frac{1-\delta_{ij}}{\nu_0^2}\right)=\frac{\mathbb{E}_{\cdot|\cdot}(\delta_{ij})}{\nu_1^2}+\frac{1-\mathbb{E}_{\cdot|\cdot}(\delta_{ij})}{\nu_0^2}:=d_{ij}^*,$$

$$\mathbb{E}_{\cdot|\cdot}\left(\frac{\gamma_q}{\sigma_1^2}+\frac{1-\gamma_q}{\sigma_0^2}\right)=\frac{\mathbb{E}_{\cdot|\cdot}(\gamma_q)}{\sigma_1^2}+\frac{1-\mathbb{E}_{\cdot|\cdot}(\gamma_q)}{\sigma_0^2}:=g_q^*.$$

Moreover, we have

$$\begin{aligned}
\mathbb{E}_{\cdot|\cdot}(z_{ij}) &= \alpha_{ij}^{(t)}+M\left(\alpha_{ij}^{(t)},1\right)\mathbb{E}_{\cdot|\cdot}(\delta_{ij})+M\left(\alpha_{ij}^{(t)},0\right)\left\{1-\mathbb{E}_{\cdot|\cdot}(\delta_{ij})\right\}\\
&= \alpha_{ij}^{(t)}+\mathbb{E}_{\cdot|\cdot}(\delta_{ij})\left\{M\left(\alpha_{ij}^{(t)},1\right)-M\left(\alpha_{ij}^{(t)},0\right)\right\}+M\left(\alpha_{ij}^{(t)},0\right),\\
\mathbb{E}_{\cdot|\cdot}(z_{ij}^2) &= \mathbb{E}_{\cdot|\cdot}(\delta_{ij})\left\{\left(\alpha_{ij}^{(t)}\right)^2+1+\alpha_{ij}^{(t)}M\left(\alpha_{ij}^{(t)},1\right)\right\}\\
&\quad+\left\{1-\mathbb{E}_{\cdot|\cdot}(\delta_{ij})\right\}\left\{\left(\alpha_{ij}^{(t)}\right)^2+1+\alpha_{ij}^{(t)}M\left(\alpha_{ij}^{(t)},0\right)\right\}\\
&= \alpha_{ij}^{(t)}\mathbb{E}_{\cdot|\cdot}(z_{ij})+1,
\end{aligned}$$

where $M(\mu,\gamma)$ is the inverse Mills ratio,

$$M(\mu,\gamma)=(-1)^{1-\gamma}\frac{\varphi(\mu)}{\Phi(\mu)^{\gamma}\left\{1-\Phi(\mu)\right\}^{1-\gamma}},\quad \mu\in\mathbb{R},\quad r=0,1,$$

$\varphi(\cdot)$ is the standard normal density and $\Phi(\cdot)$ is the standard normal cumulative distribution function. Last, since $\delta_{ij}=1$ when $z_{ij}>0$ and 0 otherwise, we always have

$$\delta_{ij}\log\mathbb{1}\{z_{ij}>0\}+(1-\delta_{ij})\log\mathbb{1}\{z_{ij}\le 0\}=0,$$

and its conditional expectation is thus zero.

The CM-step The CM-step finds the posterior mode by maximising the objective function with respect to the unknown parameters $\mathbf{\Omega}$ and $\mathbf{\Theta_1}$ in a coordinate ascent manner. The closed-form updates for the scale parameters τ_1 and τ_2 are

$$\tau_1^{(t+1)}=\frac{P(P-1)/2+2a_\tau-2}{\sum_{i<j}\omega_{ij}^2 d_{ij}^*+2b_\tau},\quad \tau_2^{(t+1)}=\frac{Q+2a_\sigma-2}{\sum_{q=1}^{Q}\beta_q^2 g_q^*+2b_\sigma}.$$

The update for ζ is

$$\zeta^{(t+1)}=\frac{2n_0+2t_0^2\sum_{i<j}\mathbb{E}_{\cdot|\cdot}(z_{ij})-2t_0^2\sum_{i<j}\sum_q\beta_q(V_{iq}+V_{jq})}{P(P-1)t_0^2+2}.$$

The update for β_q is given by

$$\begin{aligned}
\beta_q^{(t+1)} &= \frac{\sum_{i<j}(V_{iq}+V_{jq})\mathbb{E}_{\cdot|\cdot}(z_{ij})-\zeta(P-1)\sum_{i=1}^{P}V_{iq}}{(P-1)\sum_{i=1}^{P}V_{iq}^2+2\sum_{i<j}V_{iq}V_{jq}+\tau_2 g_q^*}\\
&\quad-\frac{(P-1)\sum_{i=1}^{P}V_{iq}\sum_{q'\neq q}V_{iq'}\beta_{q'}-\sum_{i<j}\sum_{q'\neq q}\beta_{q'}(V_{iq}V_{jq'}+V_{iq'}V_{jq})}{(P-1)\sum_{i=1}^{P}V_{iq}^2+2\sum_{i<j}V_{iq}V_{jq}+\tau_2 g_q^*},
\end{aligned}$$

for $q = 1, \ldots, Q$. The update for o is

$$o^{(t+1)} = \frac{\sum_{q=1}^{Q} \mathbb{E}_{\cdot|\cdot}(\gamma_q) + a_o - 1}{Q + a_o + b_o - 2}.$$

There is no closed form for the joint update of the precision matrix $\boldsymbol{\Omega}$. However, Wang (2015) provides analytical block updates, namely, by updating each column (and thus each row due to symmetry) of $\boldsymbol{\Omega}$ and iterating over all the columns,

$$\begin{aligned}
\boldsymbol{\omega}_{-ii} &= (\boldsymbol{\omega}_{i-i})^T = -\left\{ (s_{ii} + \lambda) \left(\boldsymbol{\Omega}_{-i-i}\right)^{-1} + \operatorname{diag}\left(\tau_1 \boldsymbol{d}^*_{-ii}\right) \right\}^{-1} \boldsymbol{S}_{-ii}, \\
\omega_{ii} &= \boldsymbol{\omega}_{i-i} \left(\boldsymbol{\Omega}_{-i-i}\right)^{-1} \boldsymbol{\omega}_{-ii} + \frac{N}{s_{ii} + \lambda},
\end{aligned}$$

where $\boldsymbol{S} = \boldsymbol{Y}^{\boldsymbol{T}}\boldsymbol{Y}$, $\boldsymbol{d}^*$ is a $P \times P$ matrix with entries d^*_{ij}, and the subscript $-i$ refers to the removal of ith row (the first subscript) or column (the second subscript).

1.1.2 Derivation of the variational expectation conditional maximisation algorithm

Next, we detail the VBECM algorithm for GMSS, which allows us to approximate the full parameter posterior distributions instead of targeting the posterior modes. We consider the mean-field approximation,

$$q(\boldsymbol{\Omega}, \boldsymbol{\Theta}) = q(\boldsymbol{\Omega}) \prod_{i<j} q(\delta_{ij}, z_{ij}) q(\tau) q(\zeta) \prod_{q} q(\beta_q, \gamma_q) q(o) q(\sigma^2),$$

where $\boldsymbol{\Theta} = (\boldsymbol{\delta}, \boldsymbol{z}, \tau, \zeta, \boldsymbol{\beta}, \boldsymbol{\gamma}, \sigma^2, o)$. Since the auxiliary variables z_{ij} fully determine the binary variables δ_{ij}, we group δ_{ij} and z_{ij} in one mean field factor. Then we find the optimal variational posterior by maximising the evidence lower bound (ELBO; (10) of the main text) in a coordinate ascent fashion; the optimal solution for the kth factor in the mean-field approximation, denoted by $\boldsymbol{\theta}_k$, is given by

$$q(\boldsymbol{\theta}_k) \propto \exp\left\{ \mathbb{E}_{q(\boldsymbol{\theta}_{-k})} \log p(\boldsymbol{Y}, \boldsymbol{\Omega}, \boldsymbol{\Theta}) \right\},$$

where $\boldsymbol{\theta}_{-k} = \boldsymbol{\Theta} \setminus \boldsymbol{\theta}_k$ (Bishop and Nasrabadi, 2006). The variational distribution is known in closed form except for $\boldsymbol{\Omega}$, but the conditional posterior mode of each column is known and thus can be updated by a CM-step.

The VBECM algorithm alternates between a "variational expectation step" (VBE-step), which approximates the posterior distribution (and thus the expectation) of all the parameters except $\boldsymbol{\Omega}$, and a CM-step, which evaluates the conditional maximisation for $\boldsymbol{\Omega}$, until the ELBO converges. To approximate the posterior distribution of parameters in the VBE-step, we iterate over each partition of parameters and optimise the ELBO until convergence.

The VBE-step The optimal variational distribution of δ_{ij} and z_{ij} satisfies

$$q(\delta_{ij}, z_{ij}) = q(z_{ij} \mid \delta_{ij}) q(\delta_{ij}), \quad 1 \le i < j \le P,$$

with

$$z_{ij} \mid \delta_{ij}, \boldsymbol{Y} \sim \mathcal{TN}\Big\{\alpha_{ij}^{(1)}, 1, (-1)^{1-\delta_{ij}} z_{ij} > 0\Big\}, \quad \delta_{ij} \mid \boldsymbol{Y} \sim \text{Bern}\left(\delta_{ij}^{(1)}\right),$$

where $\mathcal{TN}$ denotes a truncated normal distribution, the superscripts $^{(1)}$ and $^{(2)}$ denote the first, respectively second moments of the variable they are applied to,

$$\begin{aligned}
\alpha_{ij}^{(1)} &= \zeta^{(1)} + \sum_q V_{iq}\beta_q^{(1)} + \sum_q V_{jq}\beta_q^{(1)}, \\
\frac{1}{\delta_{ij}^{(1)}} &= 1 + \exp\left[\log\left(\frac{\nu_1}{\nu_0}\right) + \frac{\tau^{(1)}\omega_{ij}^2}{2}\left(\frac{1}{\nu_1^2} - \frac{1}{\nu_0^2}\right) + \log\Big\{1 - \Phi\left(\alpha_{ij}^{(1)}\right)\Big\} - \log\Phi\left(\alpha_{ij}^{(1)}\right)\right].
\end{aligned}$$

The first two moments of z_{ij} are

$$z_{ij}^{(1)} = \alpha_{ij}^{(1)} + M\left(\alpha_{ij}^{(1)}, 0\right) + \delta_{ij}^{(1)}\Big\{M\left(\alpha_{ij}^{(1)}, 1\right) - M\left(\alpha_{ij}^{(1)}, 0\right)\Big\}, \quad z_{ij}^{(2)} = \alpha_{ij}^{(1)} z_{ij}^{(1)} + 1.$$

Then, we find

$$\tau \mid \boldsymbol{Y} \sim \text{Gamma}(\alpha_\tau, \beta_\tau),$$

with

$$\begin{aligned}
\alpha_\tau &= \frac{P(P-1)}{4} + a_\tau, \quad \beta_\tau = \frac{1}{2}\sum_{i<j}\omega_{ij}^2\left(\frac{\delta_{ij}^{(1)}}{\nu_1^2} + \frac{1-\delta_{ij}^{(1)}}{\nu_0^2}\right) + b_\tau, \\
\tau^{(1)} &= \alpha_\tau/\beta_\tau, \quad (\log\tau)^{(1)} = \psi(\alpha_\tau) - \log(\beta_\tau),
\end{aligned}$$

where $\psi(x)$ stands for the digamma function which is defined by the gamma function and its derivative, $\psi(x) = \Gamma'(x)/\Gamma(x)$ for $x > 0$. Similarly, the optimal variational distribution of β_q and γ_q is

$$q(\beta_q, \gamma_q) = q(\beta_q \mid \gamma_q) q(\gamma_q), \quad q = 1, \ldots, Q,$$

with

$$\beta_q \mid \gamma_q, \boldsymbol{Y} = 1 \sim \mathcal{N}(\mu_{\beta,q}, \sigma_{\beta,q}^2), \quad \beta_q \mid \gamma_q, \boldsymbol{Y} = 0 \sim \delta(\beta_q), \quad \gamma_q \mid \boldsymbol{Y} \sim \text{Bern}\left(\gamma_q^{(1)}\right),$$

where

$$\begin{aligned}
\mu_{\beta,q} &= \sigma_{\beta,q}^2\Big\{\sum_{i<j}(V_{iq} + V_{jq}) z_{ij}^{(1)} - (P-1)\zeta^{(1)}\sum_{i=1}^P V_{iq} \\
&\quad -(P-1)\sum_{i=1}^P V_{iq}\sum_{q'\neq q} V_{iq'}\beta_{q'}^{(1)} - \sum_{i<j}\sum_{q'\neq q}(V_{iq}V_{jq'} + V_{iq'}V_{jq})\beta_{q'}^{(1)}\Big\}, \\
\sigma_{\beta,q}^{-2} &= \left(\sigma^{-2}\right)^{(1)} + (P-1)\sum_{i=1}^P V_{iq}^2 + 2\sum_{i<j} V_{iq}V_{jq},
\end{aligned}$$

and

$$\frac{1}{\gamma_q^{(1)}} \;=\; 1+\exp\left[\Big\{\log(1-o)\Big\}^{(1)} - \Big\{\log(o)\Big\}^{(1)} - \frac{1}{2}\left(\log\sigma^{-2}\right)^{(1)} - \frac{\mu_{\beta,q}^2}{2\sigma_{\beta,q}^2} + \frac{1}{2}\log\sigma_{\beta,q}^{-2}\right].$$

The first two moments of β_q are

$$\beta_q^{(1)} = \gamma_q^{(1)}\mu_{\beta,q}, \quad \beta_q^{(2)} = \gamma_q^{(1)}\left(\mu_{\beta,q}^2 + \sigma_{\beta,q}^2\right).$$

Also, we have

$$\zeta \mid \boldsymbol{Y} \sim \mathcal{N}(\mu_\zeta, \sigma_\zeta^2),$$

where

$$\begin{aligned} \mu_\zeta &= \sigma_\zeta^2\left\{\sum_{i<j} z_{ij}^{(1)} - \sum_q \beta_q^{(1)} \sum_{i<j}(V_{iq}+V_{jq}) + \frac{n_0}{t_0^2}\right\}, \\ \sigma_\zeta^{-2} &= \frac{1}{t_0^2} + \frac{P(P-1)}{2}, \quad \zeta^{(1)} = \mu_\zeta, \quad \zeta^{(2)} = \mu_\zeta^2 + \sigma_\zeta^2. \end{aligned}$$

The optimal variational distribution of σ^{-2} is

$$\sigma^{-2} \mid \boldsymbol{Y} \;\sim\; \text{Gamma}(\alpha_\sigma, \beta_\sigma),$$

where

$$\begin{aligned} \alpha_\sigma &= \frac{1}{2}\sum_q \gamma_q^{(1)} + a_\sigma, \quad \beta_\sigma = \frac{1}{2}\sum_q \gamma_q^{(1)}\beta_q^{(2)} + b_\sigma, \\ \left(\sigma^{-2}\right)^{(1)} &= \alpha_\sigma/\beta_\sigma, \quad \Big\{\log\left(\sigma^{-2}\right)\Big\}^{(1)} = \psi(\alpha_\sigma) - \log(\beta_\sigma). \end{aligned}$$

Finally, we find

$$o \mid \boldsymbol{Y} \sim \text{Beta}(\alpha_o, \beta_o),$$

where

$$\begin{aligned} \alpha_o &= \sum_q \gamma_q^{(1)} + a_o, \quad \beta_o = \sum_q \left(1-\gamma_q^{(1)}\right) + b_o, \quad o^{(1)} = a_o/(a_o+b_o), \\ \Big\{\log(o)\Big\}^{(1)} &= \psi(\alpha_o) - \psi(\alpha_o+\beta_o), \quad \Big\{\log(1-o)\Big\}^{(1)} = \psi(\beta_o) - \psi(\alpha_o+\beta_o). \end{aligned}$$

The CM-step The variational distribution of $\boldsymbol{\Omega}$ is not known in closed form and entails the constraint of symmetry and positive definiteness. However, the conditional distribution of its last column is tractable (Wang, 2015), allowing for conditional maximisation of the last column while holding the rest of the matrix entries fixed. We therefore can reframe this by considering the variational distribution of $\boldsymbol{\Omega}$ to a Dirac delta distribution, which is zero except for an unknown value to be estimated. This approach is equivalent to the CM-step in the ECM algorithm. For further details, we refer to the ECM derivation.

The ELBO The ELBO is given by

$$\begin{aligned}\mathcal{L}(q) &= \mathbb{E}_{q(\boldsymbol{\Omega},\boldsymbol{\Theta})}\Big\{\log p(\boldsymbol{Y},\boldsymbol{\Omega},\boldsymbol{\Theta})\Big\} - \mathbb{E}_{q(\boldsymbol{\Omega},\boldsymbol{\Theta})}\Big\{\log q(\boldsymbol{\Omega},\boldsymbol{\Theta})\Big\}\\ &= \mathcal{L}_{\boldsymbol{Y}}(\boldsymbol{Y}\mid\boldsymbol{\Omega}) + \mathcal{L}_{\boldsymbol{\Omega}}(\boldsymbol{\Omega}\mid\boldsymbol{\delta},\tau) + \sum_{i<j}\mathcal{L}_{\boldsymbol{\delta},\boldsymbol{z}}(\delta_{ij}, z_{ij}\mid\zeta,\boldsymbol{\beta}) + \mathcal{L}_{\tau}(\tau)\\ &\quad + \mathcal{L}_{\zeta}(\zeta) + \sum_{q}\mathcal{L}_{\boldsymbol{\beta},\boldsymbol{\gamma}}(\beta_q,\gamma_q\mid o,\sigma^2) + \mathcal{L}_o(o) + \mathcal{L}_{\sigma}(\sigma^2),\end{aligned}$$

where

$$\begin{aligned}\mathcal{L}_{\boldsymbol{Y}}(\boldsymbol{Y}\mid\boldsymbol{\Omega}) &= \log p(\boldsymbol{Y}\mid\boldsymbol{\Omega}) = \frac{N}{2}\log|\boldsymbol{\Omega}| - \frac{1}{2}\mathrm{tr}(\boldsymbol{Y}^T\boldsymbol{Y}\boldsymbol{\Omega}),\\ \mathcal{L}_{\boldsymbol{\Omega}}(\boldsymbol{\Omega}\mid\boldsymbol{\delta},\tau) &= \mathbb{E}_{q(\boldsymbol{\Omega},\boldsymbol{\Theta})}\Big\{\sum_{i=1}^{P}\log p(\omega_{ii}) + \sum_{i<j}\log p(\omega_{ij}\mid\delta_{ij},\tau)\Big\}\\ &= -\frac{\lambda}{2}\sum_{i=1}^{P}\omega_{ii} - \log\nu_1\sum_{i<j}\delta_{ij}^{(1)} - \log\nu_0\sum_{i<j}\left(1-\delta_{ij}^{(1)}\right)\\ &\quad -\frac{\tau^{(1)}}{2}\sum_{i<j}\omega_{ij}^2\left(\frac{\delta_{ij}^{(1)}}{\nu_1^2} + \frac{1-\delta_{ij}^{(1)}}{\nu_0^2}\right) + \frac{P(P-1)}{4}(\log\tau)^{(1)},\end{aligned}$$

$$\begin{aligned}\mathcal{L}_{\boldsymbol{\delta},\boldsymbol{z}}(\delta_{ij}, z_{ij}\mid\zeta,\boldsymbol{\beta}) &= \mathbb{E}_{q(\boldsymbol{\Omega},\boldsymbol{\Theta})}\Big\{\log p(\delta_{ij}\mid z_{ij}) + \log p(z_{ij}\mid\zeta,\boldsymbol{\beta}) - \log q(z_{ij}\mid\delta_{ij}) - \log q(\delta_{ij})\Big\}\\ &= \Big[\delta_{ij}\log\mathbb{1}\{z_{ij}\geq 0\}\Big]^{(1)} + \Big[(1-\delta_{ij})\log\mathbb{1}\{z_{ij}<0\}\Big]^{(1)}\\ &\quad -\frac{1}{2}\left(z_{ij}^{(2)} - 2\alpha_{ij}^{(1)}z_{ij}^{(1)} + \alpha_{ij}^{(2)}\right) - \mathbb{E}_{q(\boldsymbol{\Omega},\boldsymbol{\Theta})}\log q(z_{ij}\mid\delta_{ij}) - \mathbb{E}_{q(\boldsymbol{\Omega},\boldsymbol{\Theta})}\log q(\delta_{ij}),\end{aligned}$$

with

$$\begin{aligned}\mathbb{E}_{q(\boldsymbol{\Omega},\boldsymbol{\Theta})}\log q(z_{ij}\mid\delta_{ij}) &= -\frac{1}{2}\left\{z_{ij}^{(2)} - 2z_{ij}^{(1)}\alpha_{ij}^{(1)} + \left(\alpha_{ij}^{(1)}\right)^2\right\}\\ &\quad -\delta_{ij}^{(1)}\log\left\{\Phi\left(\alpha_{ij}^{(1)}\right)\right\} - \left(1-\delta_{ij}^{(1)}\right)\log\left\{1-\Phi\left(\alpha_{ij}^{(1)}\right)\right\}\\ &\quad +\delta_{ij}^{(1)}\Big[\log\mathbb{1}\{z_{ij}>0\}\Big]^{(1)} + \left(1-\delta_{ij}^{(1)}\right)\Big[\log\mathbb{1}\{z_{ij}\leq 0\}\Big]^{(1)},\end{aligned}$$

using the definition of conditional entropy and

$$\mathbb{E}_{q(\boldsymbol{\Omega},\boldsymbol{\Theta})}\log q(\delta_{ij}) = \delta_{ij}^{(1)}\log\left(\delta_{ij}^{(1)}\right) + \left(1-\delta_{ij}^{(1)}\right)\log\left(1-\delta_{ij}^{(1)}\right),$$

which is further simplified to

$$\begin{aligned}\mathcal{L}_{\boldsymbol{\delta},\boldsymbol{z}}(\delta_{ij}, z_{ij}\mid\zeta,\boldsymbol{\beta}) &= -\frac{1}{2}\alpha_{ij}^{(2)} + \frac{1}{2}\left(\alpha_{ij}^{(1)}\right)^2 + \delta_{ij}^{(1)}\log\left\{\Phi\left(\alpha_{ij}^{(1)}\right)\right\} + \left(1-\delta_{ij}^{(1)}\right)\log\left\{1-\Phi\left(\alpha_{ij}^{(1)}\right)\right\}\\ &\quad -\delta_{ij}^{(1)}\log\left(\delta_{ij}^{(1)}\right) - \left(1-\delta_{ij}^{(1)}\right)\log\left(1-\delta_{ij}^{(1)}\right).\end{aligned}$$

In addition, we have

$$\begin{aligned}
\mathcal{L}_\tau(\tau) &= \mathbb{E}_{q(\boldsymbol{\Omega},\boldsymbol{\Theta})}\Big\{\log p(\tau) - \log q(\tau)\Big\}\\
&= (a_\tau - 1)(\log\tau)^{(1)} - b_\tau\tau^{(1)} - \alpha_\tau\log\beta_\tau + \log\Gamma(\alpha_\tau) - (\alpha_\tau - 1)(\log\tau)^{(1)} + \beta_\tau\tau^{(1)}\\
&= (a_\tau - \alpha_\tau)(\log\tau)^{(1)} - (b_\tau - \beta_\tau)\tau^{(1)} - \alpha_\tau\log\beta_\tau + \log\Gamma(\alpha_\tau),\\
\mathcal{L}_\zeta(\zeta) &= \mathbb{E}_{q(\boldsymbol{\Omega},\boldsymbol{\Theta})}\Big\{\log p(\zeta) - \log q(\zeta)\Big\}\\
&= -\frac{\zeta^{(2)}}{2t_0^2} + \frac{n_0\zeta^{(1)}}{t_0^2} + \frac{1}{2}\Big\{1 + \log(2\pi\sigma_\zeta^2)\Big\},\\
\mathcal{L}_{\boldsymbol{\beta},\boldsymbol{\gamma}}(\beta_q,\gamma_q \mid \sigma^2, o) &= \mathbb{E}_{q(\boldsymbol{\Omega},\boldsymbol{\Theta})}\Big\{\log p(\beta_q \mid \gamma_q,\sigma^2) + \log p(\gamma_q \mid o) - \log q(\beta_q \mid \gamma_q) - \log q(\gamma_q)\Big\}\\
&= \left\{\gamma_q\left(\frac{1}{2}\log\sigma^{-2} - \frac{\beta_q^2}{2}\sigma^{-2}\right)\right\}^{(1)} + \Big\{(1-\gamma_q)\log\delta(\beta_q)\Big\}^{(1)}\\
&\quad + \left[\gamma_q^{(1)}(\log o)^{(1)} + \left(1 - \gamma_q^{(1)}\right)\Big\{\log(1-o)\Big\}^{(1)}\right]\\
&\quad - \mathbb{E}_{q(\boldsymbol{\Omega},\boldsymbol{\Theta})}\log q(\beta_q \mid \gamma_q) - \mathbb{E}_{q(\boldsymbol{\Omega},\boldsymbol{\Theta})}\log q(\gamma_q),
\end{aligned}$$

with

$$\begin{aligned}
\mathbb{E}_{q(\boldsymbol{\Omega},\boldsymbol{\Theta})}\log q(\beta_q \mid \gamma_q) &= -\frac{1}{2}\gamma_q^{(1)}\Big\{1 + \log\left(2\pi\sigma_{\beta,q}^2\right)\Big\} + \left(1 - \gamma_q^{(1)}\right)\Big\{\log\delta(\beta_q)\Big\}^{(1)},\\
\mathbb{E}_{q(\boldsymbol{\Omega},\boldsymbol{\Theta})}\log q(\gamma_q) &= \gamma_q^{(1)}\log\left(\gamma_q^{(1)}\right) + \left(1 - \gamma_q^{(1)}\right)\log\left(1 - \gamma_q^{(1)}\right),
\end{aligned}$$

$$\begin{aligned}
\mathcal{L}_o(o) &= \mathbb{E}_{q(\boldsymbol{\Omega},\boldsymbol{\Theta})}\Big\{\log p(o) - \log q(o)\Big\}\\
&= (a_o - 1)(\log o)^{(1)} + (b_o - 1)\Big\{\log(1-o)\Big\}^{(1)}\\
&\quad + \log B(\alpha_o,\beta_o) - (\alpha_o - 1)(\log o)^{(1)} - (\beta_o - 1)\Big\{\log(1-o)\Big\}^{(1)}\\
&= (a_o - \alpha_o)(\log o)^{(1)} + (b_o - \beta_o)\Big\{\log(1-o)\Big\}^{(1)} + \log B(\alpha_o,\beta_o),\\
\mathcal{L}_\sigma(\sigma^{-2}) &= \mathbb{E}_{q(\boldsymbol{\Omega},\boldsymbol{\Theta})}\Big\{\log p(\sigma^{-2}) - \log q(\sigma^{-2})\Big\}\\
&= (a_\sigma - 1)\left(\log\sigma^{-2}\right)^{(1)} - b_\sigma\left(\sigma^{-2}\right)^{(1)}\\
&\quad - \alpha_\sigma\log\beta_\sigma + \log\Gamma(\alpha_\sigma) - (\alpha_\sigma - 1)\left(\log\sigma^{-2}\right)^{(1)} + \beta_\sigma\left(\sigma^{-2}\right)^{(1)}\\
&= (a_\sigma - \alpha_\sigma)\left(\log\sigma^{-2}\right)^{(1)} - (b_\sigma - \beta_\sigma)\left(\sigma^{-2}\right)^{(1)} - \alpha_\sigma\log\beta_\sigma + \log\Gamma(\alpha_\sigma),
\end{aligned}$$

where $B(a,b) = \Gamma(a)\Gamma(b)/\Gamma(a+b)$ is the beta function.

Finally, we simplify the term $\alpha_{ij}^{(2)} - \left(\alpha_{ij}^{(1)}\right)^2$ in $\mathcal{L}_{\boldsymbol{\delta},\boldsymbol{z}}(\delta_{ij}, z_{ij} \mid \zeta, \boldsymbol{\beta})$, and this is identified as variance $\mathrm{Var}_{q(\boldsymbol{\Omega},\boldsymbol{\Theta})}(\alpha_{ij})$. Therefore,

$$\alpha_{ij}^{(2)} - \left(\alpha_{ij}^{(1)}\right)^2 = \mathrm{Var}_{q(\boldsymbol{\Omega},\boldsymbol{\Theta})}(\zeta) + \sum_q V_{iq}^2\mathrm{Var}_{q(\boldsymbol{\Omega},\boldsymbol{\Theta})}(\beta_q) + \sum_q V_{jq}^2\mathrm{Var}_{q(\boldsymbol{\Omega},\boldsymbol{\Theta})}(\beta_q),$$

with

$$\mathrm{Var}_{q(\boldsymbol{\Omega},\boldsymbol{\Theta})}(\zeta) \;=\; \sigma_\zeta^2, \quad \mathrm{Var}_{q(\boldsymbol{\Omega},\boldsymbol{\Theta})}(\beta_q) = \beta_q^{(2)} - \left(\beta_q^{(1)}\right)^2.$$

1.2 Hyperparameter settings

In this section, we describe the hyperparameter settings for the prior distribution of ζ. Under the simplifying assumption that no auxiliary variables are encoded, the prior distribution of edge inclusion given ζ is

$$\delta_{ij} \mid \zeta \sim \mathrm{Bern}\Big\{\Phi(\zeta)\Big\}.$$

Therefore the prior expectation and variance for the number of edges in the network are

$$\begin{aligned}
\mathbb{E}\left(\sum_{i<j}\delta_{ij}\right) &= \mathbb{E}\Bigg\{\mathbb{E}\left(\sum_{i<j}\delta_{ij} \mid \zeta\right)\Bigg\} = P\mathbb{E}\Big\{\Phi(\zeta)\Big\},\\
\mathbb{V}\left(\sum_{i<j}\delta_{ij}\right) &= \mathbb{V}\Big\{P\Phi(\zeta)\Big\} + \mathbb{E}\Big[P\Phi(\zeta)\Big\{1-\Phi(\zeta)\Big\}\Big]\\
&= \mathbb{E}\Big\{P^2\Phi(\zeta)^2\Big\} + \Big[\mathbb{E}\Big\{P\Phi(\zeta)\Big\}\Big]^2 + \mathbb{E}\Big\{P\Phi(\zeta)\Big\} - \mathbb{E}\Big\{P\Phi(\zeta)^2\Big\}\\
&= (P^2-P)\mathbb{E}\Big\{\Phi(\zeta)^2\Big\} + P\mathbb{E}\Big\{\Phi(\zeta)\Big\} + P^2\Big[E\Big\{\Phi(\zeta)\Big\}\Big]^2.
\end{aligned}$$

Next, using $\zeta \sim \mathcal{N}(n_0, t_0^2)$, we find

$$\begin{aligned}
E\Big\{\Phi(\zeta)\Big\} &= \Phi\left(\frac{n_0}{\sqrt{1+t_0^2}}\right),\\
E\Big\{\Phi(\zeta)^2\Big\} &= \Phi\left(\frac{n_0}{\sqrt{1+t_0^2}}\right) - 2T\left(\frac{n_0}{\sqrt{1+t_0^2}}, \frac{1}{\sqrt{1+t_0^2}}\right),
\end{aligned}$$

where $T(h,a)$ is Owen's T function (Owen, 1956),

$$T(h,a) \;=\; \varphi(h)\int_0^a \frac{\varphi(hx)}{1+x^2}dx, \quad a, h \in \mathbb{R},$$

with $\varphi(\cdot)$ the standard normal density function and $\Phi(\cdot)$ the standard normal cumulative density function. We thus can proceed by specifying an expectation and a variance for the prior number of edges in the network, and solving for n_0 and t_0^2.

To assess the sensitivity of this hyperprior specification, we use the "reference" simulation scenario, i.e., with $N = 200$, $P = 100$, $Q = 50$, $Q_0 = 3$. For this scenario, the average network sparsity is $\approx 3\%$, therefore the sparsity in the absence of variable-triggered hubs should be smaller. We thus evaluate the performance using a prior expectation of sparsity to 0.5%, 1% and 3%. In addition, we consider varying levels of uncertainty and set the prior standard deviation to

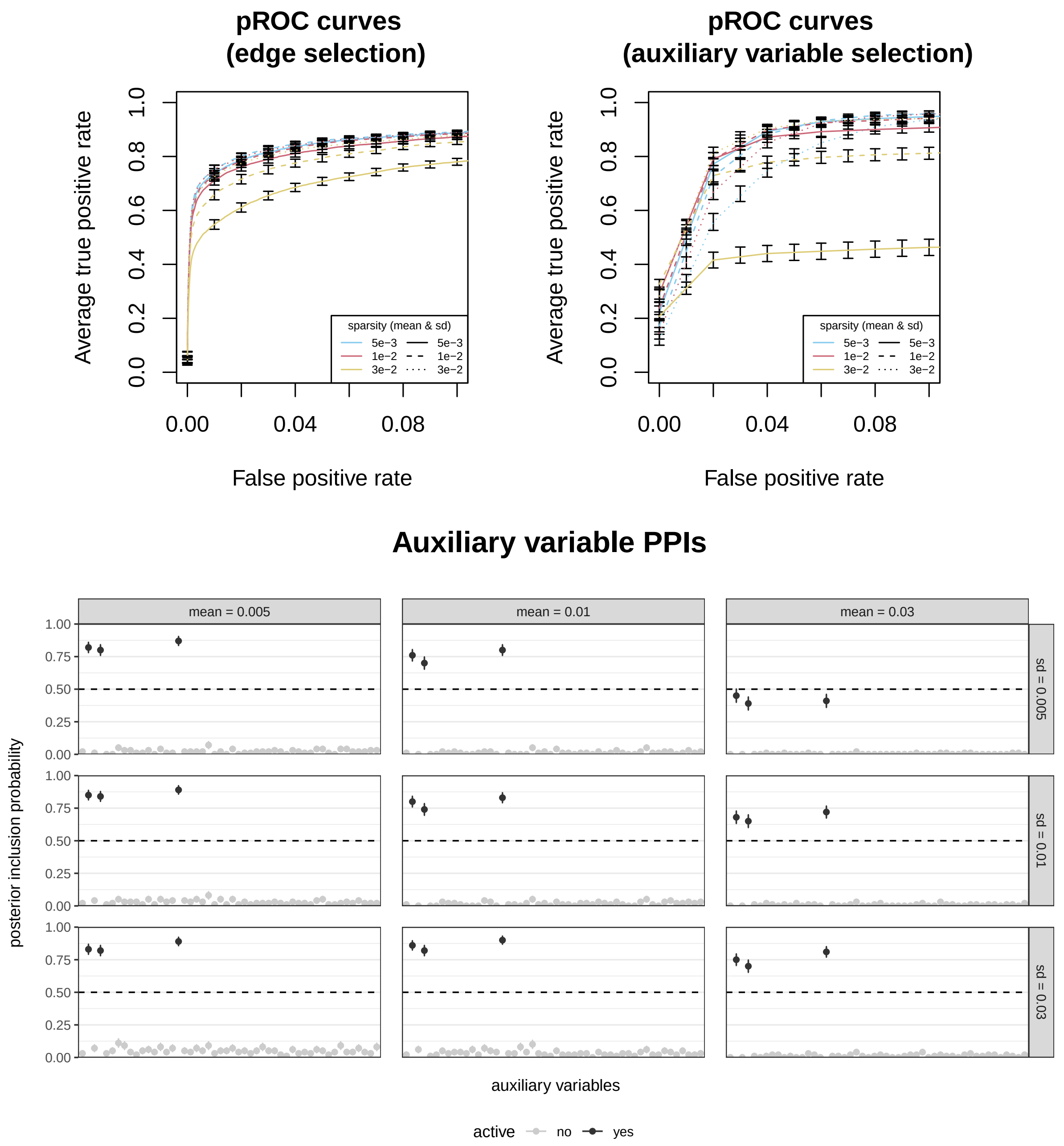

Figure 1: Sensitivity to the hyperparameter choices for ζ using GMSS on simulated data under the "reference" scenario. Top left: average pROC curves for edge selection; top right: average pROC curves for auxiliary variable selection; bottom: auxiliary variable PPIs under the hyperparameter settings of Table 1, where columns and rows correspond to different prior choices for the mean and standard deviation of the network sparsity. Error bars correspond to standard errors based on 100 replicates.

0.5%, 1% and 3% of the total number of possible edges. Table 1 summarises the hyperparameter settings considered and Figure 1 displays corresponding average partial receiver operating characteristic (pROC) curves for the edge selection and auxiliary-variable selection using GMSS. The

performance is comparable across all the hyperparameter settings. The prior expectation of 3% leads to slightly inferior performance for edge selection but large standard deviations tend to help in this scenario. In addition, active auxiliary variables are almost always effectively singled out, except in cases where the prior expectation is set too high and the standard deviation is set too low. This sensitivity study reassuringly suggests that inference is not very sensitive to reasonable guesses for these hyperparameters. In the simulations presented in the main text, we set the prior expectation to 1% and the standard deviation to 3% throughout.

Prior expectation		Prior standard deviation		Hyperparameters	
%	number	%	number	n_0	t_0^2
0.5%	25	0.5%	25	-2.69	0.09
0.5%	25	1%	50	-2.93	0.30
0.5%	25	3%	150	-4.34	1.85
1%	50	0.5%	25	-2.36	0.03
1%	50	1%	50	-2.45	0.12
1%	50	3%	150	-3.09	0.77
3%	150	0.5%	25	-1.88	0.004
3%	150	1%	50	-1.90	0.02
3%	150	3%	150	-2.04	0.18

Table 1: Prior specification for the sparsity parameter ζ, with the hyperparameters n_0 and t_0^2 derived according to the procedure presented above.

1.3 Parallel grid search procedure for spike-and-slab variances

In this section, we detail the use of model selection criteria in the grid search procedure to set the spike variance in the edge-selection bottom-level model. We compare the Akaike information criterion (AIC) adopted in the main text with the Bayesian information criterion (BIC) and the extended Bayesian information criterion (EBIC) on 100 replicates of the "reference" data generation scenario, namely, with $N = 200$ samples, $P = 100$ nodes and $Q = 50$ auxiliary variables, of which $Q_0 = 3$ contribute to the node degrees. AIC, BIC and EBIC are defined as follows

$$\begin{aligned}
\mathrm{AIC}(\nu_0) &= -N \log|\hat{\boldsymbol{\Omega}}^*| + \mathrm{tr}(\boldsymbol{Y}^T\boldsymbol{Y}\hat{\boldsymbol{\Omega}}^*) + 2\sum_{i<j} \mathbb{1}\left\{ \hat{\delta}_{ij}^{(1)} \geq 0.5 \right\},\\
\mathrm{BIC}(\nu_0) &= -N \log|\hat{\boldsymbol{\Omega}}^*| + \mathrm{tr}(\boldsymbol{Y}^T\boldsymbol{Y}\hat{\boldsymbol{\Omega}}^*) + \log(N)\sum_{i<j} \mathbb{1}\left\{ \hat{\delta}_{ij}^{(1)} \geq 0.5 \right\},\\
\mathrm{EBIC}(\nu_0) &= \mathrm{BIC}(\nu_0) + 4\gamma \log(P)\sum_{i<j} \mathbb{1}\left\{ \hat{\delta}_{ij}^{(1)} \geq 0.5 \right\},
\end{aligned}$$

where $\hat{\boldsymbol{\Omega}}$ and $\hat{\delta}_{ij}^{(1)}$ represent the posterior estimates of precision matrix and edge (i, j)'s inclusion probability, $\hat{\boldsymbol{\Omega}}^*$ refers to a thresholded precision matrix defined by

$$\hat{\omega}^*_{ij} = \begin{cases} \hat{\omega}_{ij}, & \text{if } i = j, \\ \hat{\omega}_{ij}, & \text{if } \hat{\delta}^{(1)}_{ij} \geq 0.5 \text{ and } i \neq j, \\ 0, & \text{if } \hat{\delta}^{(1)}_{ij} < 0.5 \text{ and } i \neq j, \end{cases}$$

and γ is a tuning parameter of EBIC with default choice of 0.5 (Chen and Chen, 2008).

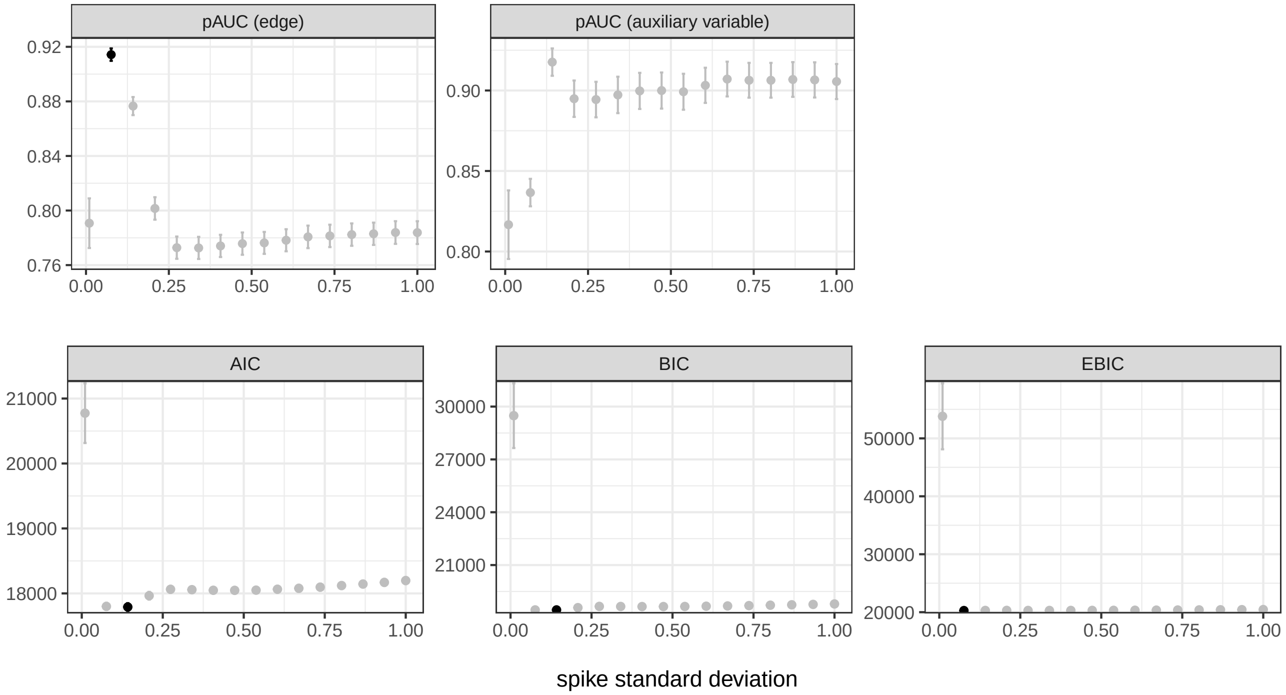

Figure 2: Spike standard deviation selection. The average partial area under the curve (pAUC) for edge and auxiliary variable selection and model selection criteria (AIC, BIC and EBIC), based on 100 replicates, are shown, for a grid of spike standard deviations ν_0. In each case, the best model, with the lowest model selection criteria or highest average pAUC for edge selection, is highlighted in black. Error bars represent standard errors.

Figure 2 compares the model selection criteria with the resulting standardised pAUCs for edge selection and auxiliary variable selection performance, for a grid of spike standard deviations. The best average pAUC is achieved at $\nu_0 = 0.07$ for edge selection, which corresponds to the lowest values of all three model selection criteria. The performance measures stabilise and remain satisfactory (pAUC > 0.8) for variable selection for $\nu_0 \geq 0.07$, while the edge selection performance deteriorates quickly after $\nu_0 = 0.07$. The values of the model selection criteria do not vary much after the smallest spike standard deviation, except for a slight upward trend in AIC after $\nu_0 = 0.07$. The three criteria point to similar models; we implemented all of them in our R package, with the AIC as a default choice.

1.4 Bayesian false discovery rate

Given posterior probabilities of inclusion, $\delta^{(1)}_{ij} = \text{pr}\,(\delta_{ij} = 1 \mid \boldsymbol{Y})$, for edges (i, j), $1 \leq i < j \leq P$, and a pre-specified threshold $\kappa \in [0, 1]$, the corresponding Bayesian false discovery rate (FDR)

can be estimated as

$$\widehat{\mathrm{FDR}}(\kappa) = \frac{\sum_{i<j} \left(1 - \delta_{ij}^{(1)}\right) \mathbb{1}\left\{\delta_{ij}^{(1)} > \kappa\right\}}{\sum_{i<j} \mathbb{1}\left\{\delta_{ij}^{(1)} > \kappa\right\}}, \tag{3}$$

following Newton et al. (2004). One can obtain a threshold corresponding to a target Bayesian FDR by interpolating a series of FDR estimates computed for a grid of thresholds $0 < \kappa_1 < \cdots < \kappa_T < 1$.

2 Addendum to the simulation experiments

2.1 Variants of the model

To ensure comparability between the GM model and the GMN and GMSS models, we introduced a variant of GM, hereafter called GM*, which replaces the beta prior on the edge inclusion probabilities by a normal prior within probit link, that is, (3) in the main text is replaced by

$$\begin{aligned} \delta_{ij} \mid \rho &\sim \text{Bern}(\rho), \\ \Phi^{-1}(\rho) &\sim \mathcal{N}(n_0, t_0^2). \end{aligned}$$

This allows us to use the same hyperprior specification procedure (Supplementary Material 1.2), and hence the same n_0 and t_0^2 for all three models, therefore putting them all on an equal footing for a fair comparison in Section 5.2 of the main text.

2.2 Edge-selection performance

We complement our illustration of the improved edge-selection performance of GMSS, compared with GM*, in the reference simulation scenario (the left panel in Figure 2B and Section 5.2 in the main text). As expected, inspecting the edges reported by GMSS but that GM* failed to detect indicates that they correspond to edges between nodes influenced by the selected auxiliary variables. Figure 3 reports the proportion of true positives among the edges reported by GMSS but not by GM* (light grey), and vice versa (dark grey) for each of the 100 data replicates. We observe that GMSS tends to detect more edges, a substantial proportion of which are true edges. In contrast, GM* generally selects fewer than 10 edges not selected by GMSS, and these are often false positives. Moreover, the posterior inclusion probabilities of edges reported by GMSS but not by GM* (light grey) are all < 0.1 in the GM* estimation but > 0.9 in the GMSS estimation.

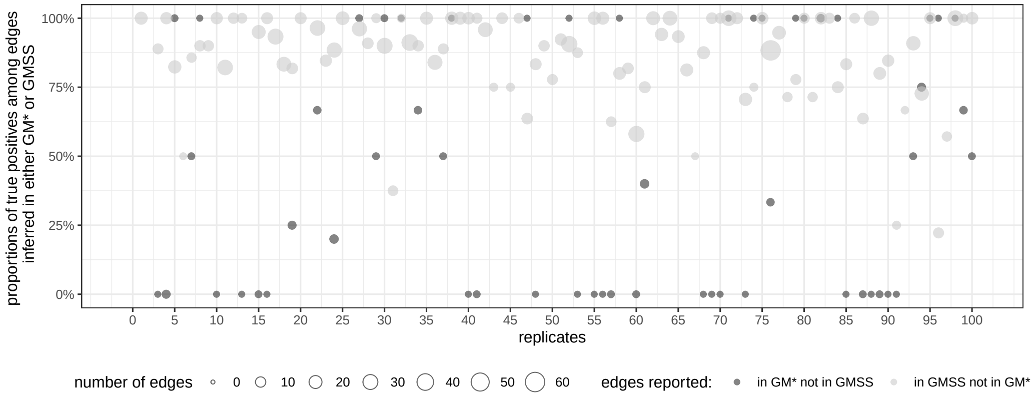

Figure 3: Proportion of true positives among edges reported by GMSS but not by GM* (light grey), and vice versa (dark grey) for each of the 100 data replicates (x-axis) of the reference simulation scenario. The size of points represents the number of edges detected exclusively by the corresponding method. Note that, for a given replicate, GMSS (resp. GM*) may not report any edge that GM* (resp. GMSS) doesn't report, in which case, no point will appear.

2.3 Non-positive effects

The numerical experiments presented in the main text (Section 5) focus on problems where auxiliary variables are associated with the *presence* of hubs, that is, thus, edges, i.e., positive effects $\boldsymbol{\beta}$ of auxiliary variables on the propensity of nodes to have high degrees. In this section, we investigate the performance of GMSS when auxiliary variables have a repressing effect on the propensity of nodes to have high degrees (negative $\boldsymbol{\beta}$, referred to as "negative scenario"), or in presence of both hub-inducing for some auxiliary variables *and* hub-repressing effects for other auxiliary variables (referred as "combined scenario").

We set ζ such that network sparsity is around 3% in both the negative and combined scenarios, with $Q_0 = 3$ and 2 active variables, respectively. In the combined scenario, the first active auxiliary variable has a positive effect and the second has a negative effect. We generate adjacency matrices, precision matrices and data following the procedure in Section 5 of the main text and for 100 data replicates.

We then apply GMSS to estimate graph structures and effects of auxiliary variables. Figure 4 indicates that GMSS is able to disentangle the active variables from the inactive ones in both scenarios. The average PPI corresponding to the negative effect of the combined scenario is somewhat lower, likely because the detection of the negative signals is being offset by the level of overall sparsity assumed.

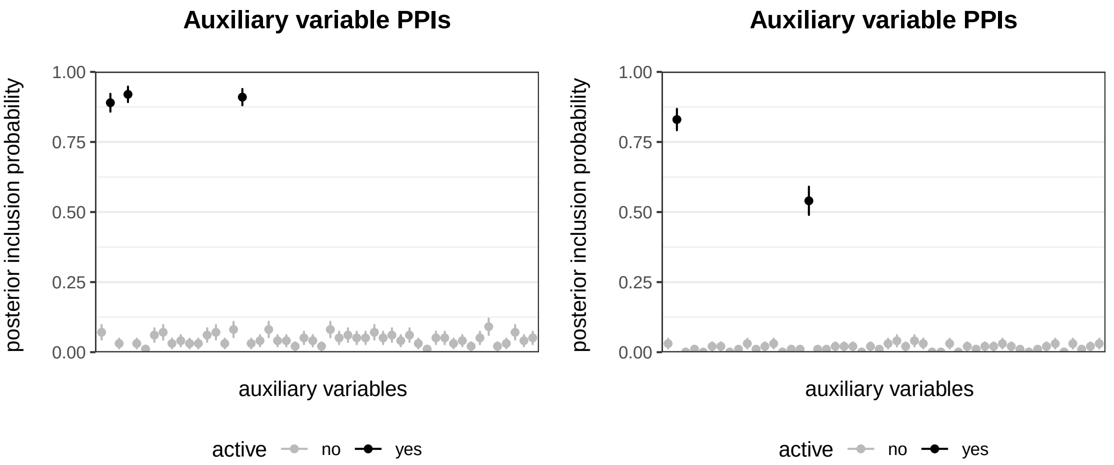

Figure 4: Performance under scenarios when node-level variables have negative effects ($Q_0 = 3$; left) and a combination of positive and negative effects ($Q_0 = 2$; right), with $N = 200$ samples, $P = 100$ nodes, $Q = 50$ candidate auxiliary variables and a network sparsity of about 3%.

2.4 Null scenario

Figure 5 presents the auxiliary variable effects estimated by GMSS and GMN, under the null-model scenario discussed in Section 5.3 of the main text, for the first replicate (left) and averaged over 100 replicates (right).

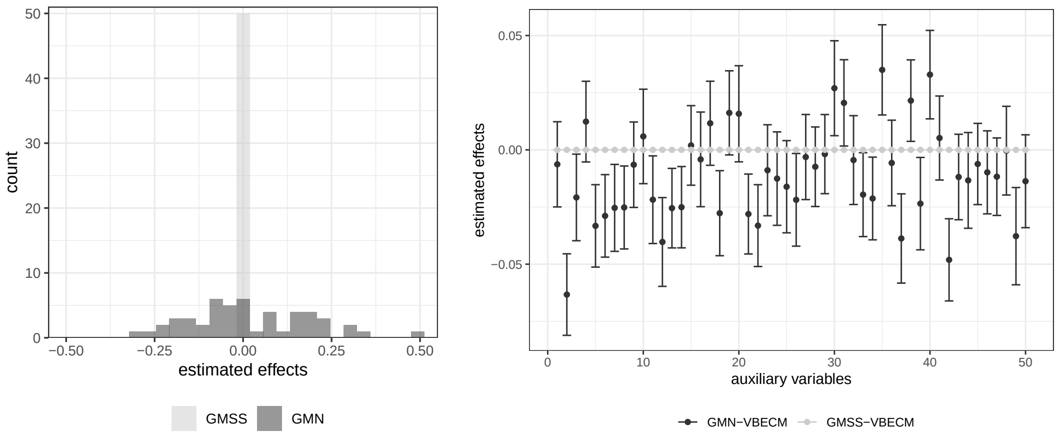

Figure 5: Auxiliary variable selection under a null-model scenario, with no active auxiliary variable. Left: histograms for effects of $Q = 50$ candidate auxiliary variables, estimated from GMN and GMSS approaches, for the first data replicate in a problem with $N = 200$ samples and $P = 100$ nodes; none of the auxiliary variables was used to simulate the graph structure. Right: average effects estimated using GMSS (grey) and GMN (black) across all 100 replicates, with standard error bars. The GMSS average effects are essentially zero, with standard error too small to be visible.

2.5 A misspecified scenario: similarity-based edge effects

In this section, we consider a simulation study where the edge model is misspecified, i.e., the assumed submodel on the edge inclusion parameter,

$$\delta_{ij} \mid \zeta, \boldsymbol{\beta} \sim \text{Bern}\left\{\Phi(\zeta + \sum_q V_{iq}\beta_q + \sum_q V_{jq}\beta_q)\right\},$$

does not reflect the data-generation mechanism. Specifically, we simulate information such that the similarity between auxiliary data for any pair of nodes influences the presence or absence of edges between the nodes. Such a setting may, for instance, be relevant in the context of brain connectivity networks, whereby nodes – quantifying fMRI signal in regions of interest – are more likely to share edges with nodes from nearby brain regions (Bu and Lederer, 2021). In this case, information about the location of these nodes may be used as auxiliary information in a similarity-based edge submodel, such as

$$\delta_{ij} \mid \zeta, \boldsymbol{\beta} \sim \text{Bern}\left[\Phi\{\zeta + \sum_q \exp(-|V_{iq} - V_{jq}|)\beta_q\}\right]. \quad (4)$$

To assess the behaviour of GMSS for such a misspecified setting, we produce 100 data replicates, as follows: we generate $Q = 50$ candidate auxiliary variables, of which 2 influence the inclusion of edges, assuming the above similarity-based edge model (4). Specifically, we use $\beta_q \neq 0$ for $q \in \{2, 44\}$, and $\beta_q = 0$ for $q \notin \{2, 44\}$. We generate the "inactive" auxiliary variables from independent standard normal distributions, and the two "active" variables from three bivariate normal distributions with different mean vectors, to reflect the fact that the nodes can be "grouped" into three distinct "regions" of a two-dimensional space. Specifically, we use the mean vectors $(-1, 0)$,

(0, 1), and (1, 0) for 30, 30 and 40 nodes, respectively. We then simulate $N = 200$ observations for $P = 100$ nodes following Section 5.1 in the main text. This procedure implies that edges are more likely between nodes located in the same "region of the space", i.e., with *similar values* of the active variables. GMSS applied to this data selects neither of the active variables (PPIs < 0.1). In other words, although the similarity between the two auxiliary features influences the edge pattern, the features themselves don't influence the propensity of nodes to have high degree, hence GMSS appropriately discards them as irrelevant to the centrality of nodes.

2.6 Handling posterior multimodality

Figure 2C and Section 5.4 of the main text illustrate the improved performance achieved by GMSS-VBECM, compared with GMSS-ECM. In this section, we investigate the possible reasons for this performance gap.

We first examine whether the performance of the ECM approach is impacted by the need for a second grid search procedure for spike-and-slab variances at the level of the auxiliary variable effects. To assess this, we consider the following top-level spike-and-slab prior formulations:

$$\begin{aligned} \beta_q \mid \gamma_q, \sigma^2 &\sim \gamma_q \mathcal{N}(0, \sigma^2) + (1 - \gamma_q)\delta(\beta_q), \quad q = 1, \dots, Q, \\ \sigma^{-2} &\sim \text{Gamma}(a_\sigma, b_\sigma), \end{aligned}$$

used in the VBECM approach, and

$$\begin{aligned} \beta_q \mid \gamma_q, \tau_2 &\sim \gamma_q \mathcal{N}(0, \tau_2^{-1}) + (1 - \gamma_q)\mathcal{N}(0, \sigma_0^2 \tau_2^{-1}), \quad q = 1, \dots, Q, \\ \tau_2 &\sim \text{Gamma}(a_\sigma, b_\sigma), \end{aligned}$$

used in the ECM approach, i.e., setting $\sigma_1 = 1$ in Equation (2) of the Supplementary Material 1.1.1. We further make the two formulations as comparable as possible by setting σ_0 to a small value, 10^{-6}, to mimic a discrete spike. Finally, we set the hyperparameters in the top-level spike-and-slab (Equation (2) in the main text) to be $\nu_1 = 100$ and $\nu_0 = 0.07$, since these choices achieve the lowest average AIC in our simulations (Figure 2). Despite these adjustments, Figure 6 below still indicates a significant performance gap between VBECM and ECM, suggesting that the performance gap may not arise from the "double grid search" nor a discrete formulation of spike-and-slab, but rather from the inference algorithms themselves.

We next investigate the potential ability of variational inference to mitigate entrapment in local modes. We expect that this may result from approximating of full posterior distributions, unlike with the ECM algorithm. Specifically, using the structured mean-field approximation of Equation (11) of the main text retains a joint distribution for the spike-and-slab parameters β_q and γ_q. To explore this, we consider one data replicate and run the two inference algorithms using 200 different random starts. Figure 2C of the main text displays the obtained optimal values for the objective functions (Q function for the ECM algorithm and ELBO for the VBECM algorithm): the Q function values obtained by the ECM algorithm exhibit high variability, suggesting that it reaches different local optima. In contrast, the ELBO values reached by the VBECM algorithm are consistently high across the random starts.

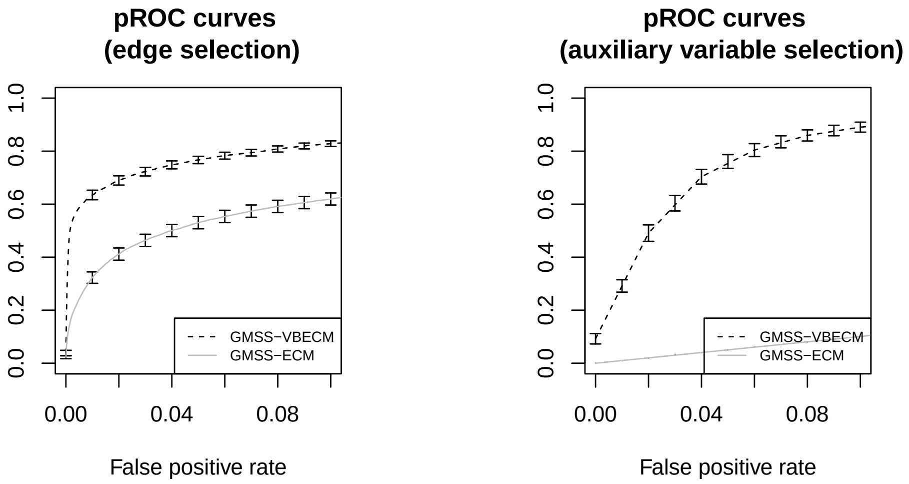

Figure 6: Comparison between VBECM and ECM inference for the GMSS model for a given spike-and-slab prior configuration. Average pROC curves for edge selection (left) and auxiliary variable selection (right) using GMSS, along with standard error bars based on 100 replicates. We consider spike-and-slab priors with $\nu_1 = 100, \nu_0 = 0.07, \sigma_1 = 1, \sigma_0 = 10^{-6}$.

2.7 Variational credible intervals

In this section, we illustrate the advantages of quantifying uncertainty around the estimates using variational inference for the GMSS model. We simulate $N = 100$ independent samples from a $P = 20$ dimensional multivariate normal distribution, whose precision matrix structure is influenced by one auxiliary variable out of 10 candidate variables. We set the overall network sparsity to be 1%, i.e., $\zeta = \Phi^{-1}(0.01)$. The effect of the fifth variable is set to 2, and all other effects are set to 0. For each replicate, we use the procedure described in Section 5.1 of the main text to generate auxiliary variables and simulate the adjacency matrix such that $A_{ij} \sim \text{Bern}\{\Phi(\zeta + 2V_{i5} + 2V_{j5})\}$, for $1 \leq i < j \leq P$. The precision matrix and data are generated as described in Section 5.1 of the main text.

We compare the estimations of the effects of auxiliary variables obtained with GMSS-ECM and GMSS-VBECM. Figure 7 shows that the ECM algorithm does not always produce null estimates for the inactive auxiliary variables (i.e., all but the fifth), while the variational credibility bars produced by the VBECM algorithm cover zero in more than 95% of the cases. For the active auxiliary variable (the fifth variable), the VBECM algorithm also tends to provide better estimates compared to the ECM algorithm, although the coverage of the variational credible intervals sometimes fail to cover the true value. This is likely because of the tendency of variational inference to underestimate posterior variances. This is a well-known drawback resulting from optimising the *reverse* Kullback–Leibler divergence (which tends to prevent the approximation from putting mass in region of the parameter space where the true distribution has little mass) as well as from the use of factorised mean-field distributions. The sparse prior specification, here the spike-and-slab prior, likely results in further unwanted shrinkage towards zero. However, it is important to note that our VBECM algorithm clearly outperforms the ECM implementation for variable selection, which, rather than effect size estimation, remains our primary goal.

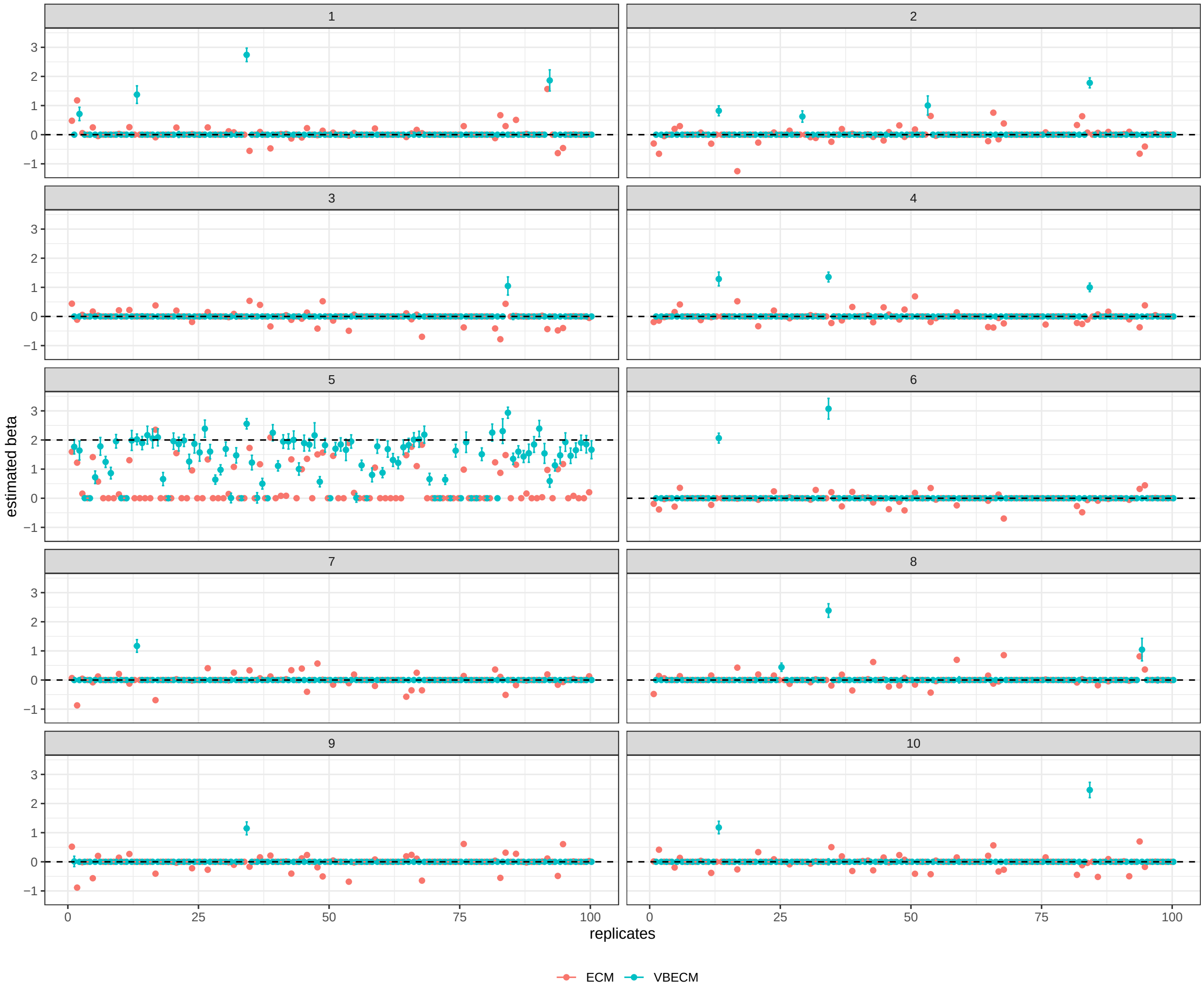

Figure 7: Auxiliary effect estimates by GMSS-ECM (red) and GMSS-VBECM (blue) in an example with $N = 100$ samples, $P = 20$ nodes and $Q = 10$ candidate auxiliary variables. Each panel represents one auxiliary variable and the true, simulated effects are highlighted by the dashed horizontal lines. The estimated effects using GMSS-ECM and GMSS-VBECM are shown for 100 replicates (x-axis). Error bars for the VBECM algorithm correspond to the 95% variational credible intervals (unavailable for the ECM algorithm).

2.8 Runtime profiling

We next present the runtime profiling for all simulation scenarios of Table 1 of the main text. Table 2 indicates that, although the GM approach is the fastest due to its simpler model with no encoding of auxiliary variables, the top-level spike-and-slab specification of the GMSS approach permits saving about two-third of the computational time used by the GMN approach. The GMSS runs complete in less than or about one minute on average, across all the data scenarios.

	Sparsity	Noise	Q_0	Q	N	P	GM*	GMN	GMSS
1.	3%	10%	3	50	200	100	3.56 (0.17)	63.63 (1.92)	41.69 (1.27)
2.	3%	10%	3	50	200	50	0.48 (0.02)	4.97 (0.12)	5.02 (0.24)
3.	3%	10%	3	50	100	100	14.66 (0.21)	167.71 (6.42)	51.94 (1.06)
4.	3%	10%	3	50	100	50	0.84 (0.01)	5.92 (0.10)	5.69 (0.15)
5.	3%	10%	3	20	200	100	3.40 (0.17)	35.09 (0.78)	22.53 (0.64)
6.	3%	10%	3	100	200	100	3.51 (0.17)	73.71 (2.73)	60.73 (1.63)
7.	3%	10%	1	50	200	100	2.96 (0.09)	57.75 (1.36)	52.17 (1.23)
8.	3%	10%	5	50	200	100	3.35 (0.17)	61.07 (1.75)	30.30 (0.83)
9.	3%	20%	3	50	200	100	3.44 (0.17)	57.38 (2.09)	35.53 (1.08)
10.	3%	30%	3	50	200	100	3.26 (0.15)	56.78 (1.86)	35.47 (0.88)
11.	1%	8.5%	3	50	200	100	3.13 (0.16)	48.28 (1.41)	32.80 (1.07)
12.	10%	8.5%	3	50	200	100	6.07 (0.22)	69.81 (2.76)	29.47 (0.82)

Table 2: Average runtime, including grid search, in seconds for GM*, GMN and GMSS in the simulation experiments presented in Table 1 (main text) on an Intel Xeon CPU, 2.60 GHz. Standard errors based on 100 replicates are in parentheses.

3 Addendum to the monocyte network application

Table 3 presents hub genes in the monocyte networks inferred by both GM* and GMSS methods. Table 4 and Table 5 list the neighbours of the *LYZ*, *YEATS4* and *CREB1* identified using the GM* and GMSS approaches, for unstimulated and stimulated monocyte data respectively.

unstimulated					stimulated				
GM*		GMSS			GM*		GMSS		
Gene	Degree	Gene	Degree	Change	Gene	Degree	Gene	Degree	Change
TRIM16L	24	*TRIM16L*	27	3	*TP53BP2*	22	*TP53BP2*	26	4
TP53BP2	22	*CHRNA5*	25	7	*CDKN2AIPNL*	21	*ZMAT3*	24	4
CDKN2AIPNL	19	*TP53BP2*	24	2	*CCBE1*	20	*CCBE1*	23	3
PPID	19	***CREB1***	20	2	*TRIM16L*	20	*CDKN2AIPNL*	23	2
CHRNA5	18	*CDKN2AIPNL*	19	0	*ZMAT3*	20	*KCNH6*	21	5
CREB1	18	*PPID**	19	0	*BLZF1*	19	*TRIM16L*	20	0
CCBE1	17	*CCBE1*	18	1	*KIAA0101*	19	*ZNF266*	20	4
KCNH6	17	*ZNF394*	18	4	***CREB1***	18	*BLZF1**	19	0
ZMAT3	17	*KCNH6*	17	0	*CHRNA5*	17	*KIAA0101*	19	0
FKBP14	16	*KIAA0101*	17	3	*MCF2L2*	17	*MCF2L2*	19	2
ZNF682	16	*FKBP14**	16	0	*NDUFV3*	17	***CREB1***	18	0
DDX51	14	*ZMAT3*	16	-1	*PPID*	17	*SNRNP48*	18	1
KIAA0101	14	*ZNF682**	16	0	*SNRNP48*	17	*CHRNA5*	17	0
USP49	14	*AIRE*	15	2	*KCNH6*	16	*NDUFV3*	17	0
ZNF394	14				*ZNF266*	16			
ZNF738	14								

Table 3: Genes with node degrees larger than the 90th percentile in the GM* and GMSS networks, with the difference in degree between the two networks, for unstimulated (left) and stimulated (right) monocyte analyses. The genes not controlled by the active auxiliary variables are marked with * in the GMSS network. The genes suspected to be genetic mediators (Ruffieux et al., 2020, 2021) are highlighted in bold.

LYZ					YEATS4					CREB1				
GM*		GMSS			GM*		GMSS			GM*		GMSS		
Gene	Degree	Gene	Degree	Change	Gene	Degree	Gene	Degree	Change	Gene	Degree	Gene	Degree	Change
TRIM16L	24	TRIM16L	27	3	TP53BP2	22	TP53BP2	24	2	TRIM16L	24	TRIM16L	27	3
TP53BP2	22	CHRNA5	25	7	PPID	19	KCNH6	17	0	CDKN2AIPNL	19	CHRNA5	25	7
**CREB1**	18	TP53BP2	24	2	KCNH6	17	USP49	13	-1	CHRNA5	18	CDKN2AIPNL	19	0
KCNH6	17	**CREB1**	20	2	USP49	14	TAF15	6	0	CCBE1	17	CCBE1	18	1
KIAA0101	14	KCNH6	17	0	TAF15	6				KCNH6	17	ZNF394	18	4
SEMA3E	13	KIAA0101	17	3						ZMAT3	17	KCNH6	17	0
ZNF69	9	SEMA3E	14	1						FKBP14	16	KIAA0101	17	3
NDUFV3	7	ZNF69*	9	0						DDX51	14	FKBP14*	16	0
		NDUFV3*	7	0						KIAA0101	14	ZMAT3	16	-1
										ZNF394	14	AIRE	15	2
										AIRE	13	SEMA3E	14	1
										SEMA3E	13	BLZF1*	13	1
										BLZF1	12	LOC729603	13	5
										LRRFIP1	12	DDX51*	11	-3
										**LYZ**	9	**LYZ***	10	1
										ZNF430	8	LRRFIP1*	9	-3
										RAB27A	4	ZNF430	8	0
												ZNF786*	6	1
												RAB27A	5	1

Table 4: Neighbours of *LYZ*, *YEATS4* and *CREB1* ranked by their degrees as estimated by GM* and GMSS, with difference in degree between the two *unstimulated* networks. *LYZ*, *YEATS4* and *CREB1* are highlighted in bold. The genes not controlled by active auxiliary variables at 20% FDR are marked with * for the GMSS network.

LYZ					YEATS4					CREB1				
GM*		GMSS			GM*		GMSS			GM*		GMSS		
Gene	Degree	Gene	Degree	Change	Gene	Degree	Gene	Degree	Change	Gene	Degree	Gene	Degree	Change
TP53BP2	22	TP53BP2	26	4	TP53BP2	22	TP53BP2	26	4	CDKN2AIPNL	21	CCBE1	23	3
ZMAT3	20	ZMAT3	24	4	MCF2L2	17	MCF2L2	19	2	CCBE1	20	CDKN2AIPNL	23	2
**CREB1**	18	KCNH6	21	5	TAF15	6	TAF15	6	0	TRIM16L	20	TRIM16L	20	0
CHRNA5	17	**CREB1**	18	0						BLZF1	19	BLZF1*	19	0
NDUFV3	17	CHRNA5	17	0						KIAA0101	19	KIAA0101	19	0
SEMA3E	14	NDUFV3	17	0						CHRNA5	17	CHRNA5	17	0
TRIM34	12	TRIM34	16	4						NDUFV3	17	NDUFV3	17	0
TMEM106A	9	SEMA3E	14	0						SEMA3E	14	TRIM34	16	4
TNFSF14	6	C19orf12	9	1						LRRFIP1	13	LOC729603	15	3
ZNF131	2	TMEM106A	8	-1						LOC729603	12	QRFPR	14	2
		TNFSF14	6	0						QRFPR	12	SEMA3E	14	0
		ZNF131	3	1						TRIM34	12	BMS1P5	13	2
										BMS1P5	11	**LYZ***	13	2
										**LYZ**	11	LRRFIP1	12	-1
										USP49	10	USP49	10	0
										TMEM106A	9	TMEM106A	8	-1
										HNRNPU	4	PPM1K*	4	1

Table 5: Neighbours of *LYZ*, *YEATS4* and *CREB1* ranked by their degrees as estimated by GM* and GMSS, with difference in degree between the two *stimulated* networks. *LYZ*, *YEATS4* and *CREB1* are highlighted in bold. The genes not controlled by active auxiliary variables at 20% FDR are marked with * for the GMSS network.

References

Bishop, C. M. and Nasrabadi, N. M. (2006). *Pattern recognition and machine learning*, volume 4. Springer.

Bu, Y. and Lederer, J. (2021). Integrating additional knowledge into the estimation of graphical models. *The international journal of biostatistics*, 18(1):1–17.

Chen, J. and Chen, Z. (2008). Extended Bayesian information criteria for model selection with large model spaces. *Biometrika*, 95(3):759–771.

Li, Z. R. and McCormick, T. H. (2019). An expectation conditional maximization approach for Gaussian graphical models. *Journal of Computational and Graphical Statistics*, 28(4):767–777.

Meng, X.-L. and Rubin, D. B. (1993). Maximum likelihood estimation via the ECM algorithm: A general framework. *Biometrika*, 80(2):267–278.

Newton, M. A., Noueiry, A., Sarkar, D., and Ahlquist, P. (2004). Detecting differential gene expression with a semiparametric hierarchical mixture method. *Biostatistics*, 5:155–176.

Owen, D. B. (1956). Tables for computing bivariate normal probabilities. *The Annals of Mathematical Statistics*, 27(4):1075–1090.

Ruffieux, H., Davison, A. C., Hager, J., Inshaw, J., Fairfax, B. P., Richardson, S., and Bottolo, L. (2020). A global-local approach for detecting hotspots in multiple-response regression. *The Annals of Applied Statistics*, 14(2):905.

Ruffieux, H., Fairfax, B. P., Nassiri, I., Vigorito, E., Wallace, C., Richardson, S., and Bottolo, L. (2021). EPISPOT: an epigenome-driven approach for detecting and interpreting hotspots in molecular QTL studies. *The American Journal of Human Genetics*, 108(6):983–1000.

Wang, H. (2015). Scaling it up: Stochastic search structure learning in graphical models. *Bayesian Analysis*, 10(2):351–377.
